# The HIV-1 Env gp120 Inner Domain Shapes the Phe43 Cavity and the CD4 Binding Site

**DOI:** 10.1128/mBio.00280-20

**Published:** 2020-05-26

**Authors:** Jérémie Prévost, William D. Tolbert, Halima Medjahed, Rebekah T. Sherburn, Navid Madani, Daria Zoubchenok, Gabrielle Gendron-Lepage, Althea E. Gaffney, Melissa C. Grenier, Sharon Kirk, Natasha Vergara, Changze Han, Brendan T. Mann, Agnès L. Chénine, Adel Ahmed, Irwin Chaiken, Frank Kirchhoff, Beatrice H. Hahn, Hillel Haim, Cameron F. Abrams, Amos B. Smith, Joseph Sodroski, Marzena Pazgier, Andrés Finzi

**Affiliations:** a Centre de Recherche du CHUM, Montreal, Quebec, Canada; b Département de Microbiologie, Infectiologie et Immunologie, Université de Montréal, Montreal, Quebec, Canada; c Infectious Diseases Division, Department of Medicine of Uniformed Services, University of the Health Sciences, Bethesda, Maryland, USA; d Department of Cancer Immunology and Virology, Dana-Farber Cancer Institute, Boston, Massachusetts, USA; e Department of Microbiology, Harvard Medical School, Boston, Massachusetts, USA; f Department of Chemistry, University of Pennsylvania, Philadelphia, Pennsylvania, USA; g U.S. Military HIV Research Program, Walter Reed Army Institute of Research, Silver Spring, Maryland, USA; h Henry M. Jackson Foundation for the Advancement of the Military Medicine, Bethesda, Maryland, USA; i Department of Biochemistry and Molecular Biology, Drexel University College of Medicine, Philadelphia, Pennsylvania, USA; j Institute of Molecular Virology, Ulm University Medical Center, Ulm, Germany; k Departments of Medicine and Microbiology, Perelman School of Medicine, University of Pennsylvania, Philadelphia, Pennsylvania, USA; l Department of Microbiology, Carver College of Medicine, University of Iowa, Iowa City, Iowa, USA; m Department of Microbiology and Immunology, McGill University, Montreal, Quebec, Canada; Vaccine Research Center, National Institute of Allergy and Infectious Diseases; University of Pittsburgh School of Medicine

**Keywords:** Env, gp120, CD4, CD4mc, CRF01_AE, ADCC, CD4 binding site, CD4 mimetic, HIV-1, Phe43 cavity, neutralization

## Abstract

The HIV-1 envelope glycoproteins (Env) undergo conformational changes upon interaction of the gp120 exterior glycoprotein with the CD4 receptor. The gp120 inner domain topological layers facilitate the transition of Env to the CD4-bound conformation. CD4 engages gp120 by introducing its phenylalanine 43 (Phe43) in a cavity (“the Phe43 cavity”) located at the interface between the inner and outer gp120 domains. Small CD4-mimetic compounds (CD4mc) can bind within the Phe43 cavity and trigger conformational changes similar to those induced by CD4. Crystal structures of CD4mc in complex with a modified CRF01_AE gp120 core revealed the importance of these gp120 inner domain layers in stabilizing the Phe43 cavity and shaping the CD4 binding site. Our studies reveal a complex interplay between the gp120 inner domain and the Phe43 cavity and generate useful information for the development of more-potent CD4mc.

## Abstract (precis)

The Phe43 cavity of HIV-1 envelope glycoproteins (Env) is an attractive druggable target. New promising compounds, including small CD4 mimetics (CD4mc), were shown to insert deeply into this cavity. Here, we identify a new network of residues that helps to shape this highly conserved CD4 binding pocket and characterize the structural determinants responsible for Env sensitivity to small CD4 mimetics.

## INTRODUCTION

The human immunodeficiency virus type 1 (HIV-1) envelope glycoproteins (Env) mediate virus entry into host cells to initiate the viral replication cycle. The gp120 exterior Env subunit mediates the initial interaction with the CD4 receptor. The gp120 phenylalanine 43 (Phe43) cavity is a conserved region of ∼150 Å^3^ where Phe43 of CD4 makes numerous contacts with conserved gp120 residues and is connected with the gp120 inner domain and the coreceptor binding site via a water-filled solvent channel (1, 2). This interaction triggers major conformational changes allowing coreceptor (i.e., CCR5 and CXCR4) binding (3–10). Subsequent conformational changes in gp41 lead to the formation of the six-helix bundle, resulting in the fusion of the viral envelope and the target cell membrane (11, 12). CD4-induced (CD4i) conformational changes in the gp120 inner domain involve three flexible topological layers (layers 1, 2, and 3). Despite a lack of contact with CD4, the gp120 inner domain layers govern CD4 triggering by participating in conformational transitions within gp120 and regulating the interaction with gp41 (13, 14). Structural rearrangements between layer 1 and layer 2 were previously shown to facilitate the transition of the envelope glycoprotein trimer from the unliganded state to the CD4-bound state and to stabilize gp120-CD4 interactions (14–16). Layer 3 was previously shown to govern the efficiency of the initial gp120 interaction with CD4, a function that can also be fulfilled by filling the Phe43 cavity with a bulky residue at position 375 (13, 14, 17).

Env represents the only viral antigen exposed on the surface of virions and infected cells and thus is the primary target for antibodies (Abs). Despite targeting different epitopes, most broadly neutralizing antibodies (bNAbs) preferentially recognize the “closed” conformation of Env (18, 19). Conversely, CD4 binding forces Env to adopt “open” conformations (20, 21), allowing its recognition by nonneutralizing Abs (nnAbs) (22, 23). Env-CD4 complexes at the surface of HIV-1-infected cells were previously shown to be efficiently recognized by CD4-induced (CD4i) Abs, which are present in sera from HIV-1-infected individuals and can mediate potent antibody-dependent cellular cytotoxicity (ADCC) responses (24–28). HIV-1 evolved to minimize the exposure of these CD4i epitopes by limiting Env-CD4 interaction. HIV-1 accomplishes this through its Nef and Vpu accessory proteins, which decrease the overall amounts of Env (via Vpu-mediated BST-2 downregulation) and CD4 at the cell surface (23, 29, 30). In addition, efficient Env internalization also limits ADCC responses (31, 32). Motivated by the vulnerability of the CD4-bound conformation of Env to ADCC, new approaches to “force” Env to adopt this conformation using small CD4-mimetic compounds (CD4mc) have been developed (33). The antiviral activity of CD4mc is not limited to ADCC; these compounds can compete for Env-CD4 interaction, mediate viral particle inactivation by prematurely triggering Env, and sensitize infectious viral particles to neutralization by otherwise nnAbs (34–37). The potential clinical benefit of CD4mc was highlighted in two recent *in vivo* studies showing how CD4mc can act as prophylactic agents to decrease HIV-1 acquisition in humanized mice and simian-human immunodeficiency virus (SHIV)-challenged nonhuman primates (NHP) (38, 39).

Interestingly, Env transitions to the CD4-bound conformation can be modulated by single-residue substitutions. For example, the replacement of the well-conserved group M serine at position 375 by a large hydrophobic residue, such as tryptophan, fills the Phe43 cavity; this substitution alters Env conformation by predisposing gp120 to spontaneously assume a state closer to the CD4-bound conformation (13, 14, 40). While S375 is well conserved in the majority of group M HIV-1 isolates, CRF01_AE Env possesses a Phe43 cavity-filling residue at position 375 (H375) (41–43). The presence of H375 was linked to the natural exposure of CD4i epitopes in CRF01_AE strains, resulting in their enhanced susceptibility to ADCC responses (42). Besides modulating Env interaction with human CD4 (41), residue 375 was shown to modulate SHIV binding to rhesus monkey CD4 and replication in nonhuman primates (44), highlighting its critical role in viral pathogenesis. By performing structural, *in silico*, and functional analyses, using CD4mc as probes, we uncovered how the gp120 inner domain shapes the Phe43 cavity and the CD4 binding site.

## RESULTS

### Comparison of Phe43 cavity and coevolving inner domain layer residues among HIV-1 clades.

**FIG 1:**
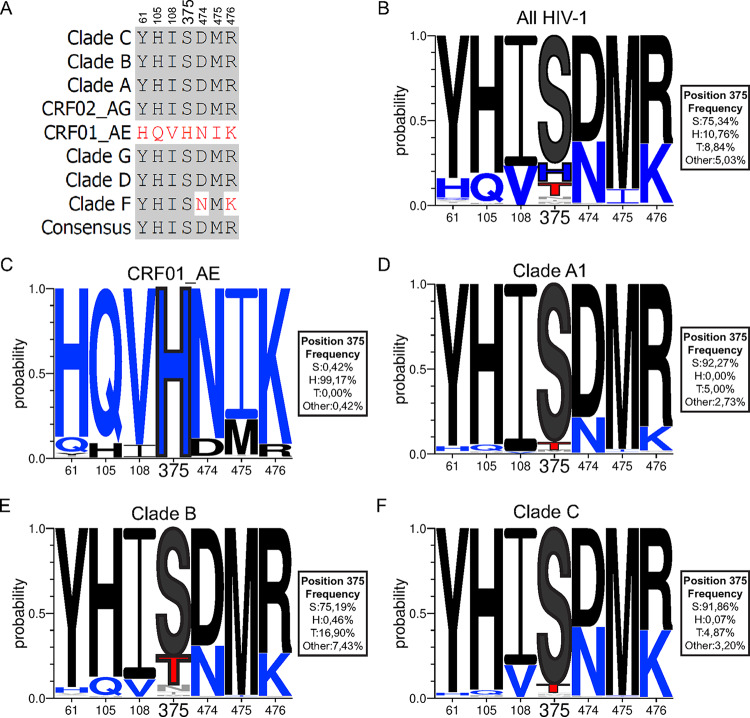
Sequence alignment of gp120 Phe43 cavity and inner domain layer residues of different HIV-1 isolates. (A) Sequence alignment of selected gp120 residues located in the Phe43 cavity (375) or the inner domain layers (61, 105, 108, 474, 475, and 476) based on the Env consensus sequence of each of the HIV-1 group M major subtypes (clades A, B, C, D, F, G, CRF01_AE, and CRF02_AG). The 2017 Los Alamos database-curated Env alignment was used as the basis for this figure, which contains 5,471 amino acid HIV-1 group M sequences (including 481 sequences of CRF01_AE, 220 of subtype A1, 1,937 of subtype B, and 1,377 of subtype C). Residue numbering is based on that of the HXBc2 strain of HIV-1. Identical residues are shaded in dark gray, and nonidentical residues are highlighted in red. (B to F) Logo depictions of the frequency of each amino acid from the Phe43 cavity at positions 61, 105, 108, 375, 474, 475, and 476 in all HIV-1 isolates (B) or in isolates from CRF01_AE (C), clade A1 (D), clade B (E), and clade C (F). The height of the letter indicates its frequency within the clade. S375 and its coevolving residues are shown in black, H375 and its coevolving residues are shown in blue, and T375 is shown in red. The box beside each logo indicates the frequency of all the amino acids at position 375.

We previously reported that six residues within the gp120 inner domain layers coevolved with Phe43 cavity residue 375 to facilitate CD4 interaction (41). We analyzed all available HIV-1 sequences together or segregated by clades using the NIH Los Alamos HIV database to determine the degree of conservation of these residues located in layer 1 (residue 61), layer 2 (residues 105 and 108), and layer 3 (residues 474, 475, and 476) (collectively named LM, for layer mutants) and in the Phe43 cavity (residue 375). The consensus sequences of these residues from CRF01_AE strains diverge from those of all other major HIV-1 subtypes (clades A, B, C, D, F, G, and CRF02_AG) (Fig. 1A). Among the coevolving inner domain residues, most of the clades (except clade F [N474 and K476]) share the same consensus sequence (Y61, H105, I108, D474, M475, and R476), which differs from that seen with the residues found in CRF01_AE strains (H/Q61, Q105, V108, N474, I475, K476). Serine 375 is the predominant residue (>75%) in all HIV-1 major subtypes, except for CRF01_AE (Fig. 1A to C). Other residues were found to occupy position 375 (T375, N375, I375, and M375), with T375 being present in more than 8% of all HIV-1 strains (Fig. 1B). T375 is present in clade B (16.9%), clade A1 (5%), and clade C (4.87%) but also in clade D (7.76%), CRF02_AG (5.26%), and clade F (2.27%). Interestingly, CRF01_AE strains have a highly conserved histidine at position 375 (H375; >99%) (41–43).

### Phe43 cavity and gp120 inner domain layer changes render CRF01_AE strains susceptible to CD4mc antiviral activities.

**FIG 2:**
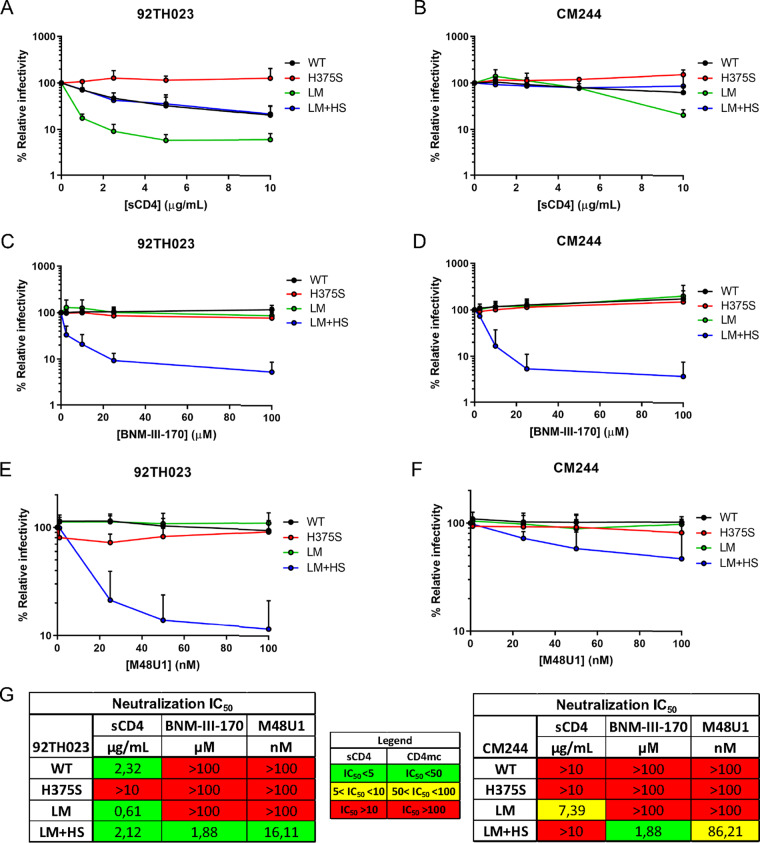
Effect of gp120 layer mutations on neutralization by CD4 and CD4mc. Recombinant HIV-1 strains expressing luciferase and bearing wild-type or mutant CRF01_AE Envs (92TH023 and CM244 isolates) were normalized by reverse transcriptase activity. Normalized amounts of viruses were incubated with serial dilutions of sCD4 (A and B), BNM-III-170 (C and D), or M48U1 (E and F) at 37°C for 1 h prior to infection of Cf2Th-CD4/CCR5 cells. Infectivity at each dilution of sCD4 or CD4mc tested is shown as the percentage of infection without sCD4 or CD4mc for each particular mutant. Quadruplicate samples were analyzed in each experiment. Data shown are the means of results obtained in at least 3 independent experiments. The error bars represent the standard deviations. Neutralization half-maximal inhibitory concentration (IC_50_) values are summarized in panel G.

To evaluate the potential impact of the LM residues on shaping the Phe43 cavity, we decided to use CD4mc as probes. Several cocrystals of different CD4 mimics and gp120 cores exist, providing detailed structural information regarding their mode of interaction within the cavity (1, 37, 45–51). Briefly, both CD4mc (molecular weight [MW], ∼500 Da) and CD4 miniproteins (∼3 kDa) were optimized to display a large hydrophobic group (phenyl or cyclohexyl ring) that projects deeply into the Phe43 cavity, allowing them to reach the cavity edge where residue 375 is located. These projections were found to go deeper into the Phe43 cavity than CD4 Phe43 residue itself, rendering these molecules more sensitive to changes occurring within the cavity. It has been previously reported that the presence of bulky cavity-filling residues at this position (H375, W375, Y375, R375) confers resistance to CD4mc and CD4 miniproteins by abrogating their interaction deep within the Phe43 cavity (34, 36, 47, 48, 50, 52, 53). Replacement of the naturally occurring CRF01_AE H375 with a serine residue (H375S) has been shown to reduce CD4 binding. Introduction of the LM substitutions (H/Q61Y, Q105H, V108I, N474D, I475M, and K476R) restored this interaction (41). However, whether these Phe43 cavity and inner domain changes affect CD4mc sensitivity has not yet been determined. First, we evaluated the effect of the H375S change on the sensitivity of two CRF01_AE isolates (tier 1 92TH023 and tier 2 CM244) to neutralization by different CD4 mimics, including soluble CD4 (sCD4), CD4mc (BNM-III-170), and a CD4 miniprotein (M48U1) (33, 54). In agreement with previous results (41), replacement of histidine 375 by a serine residue (H375S) in both HIV-1_CRF01_AE_ Envs completely abolished the susceptibility of pseudotyped virions to sCD4 neutralization (Fig. 2A and B to G). Viral particles bearing both CRF01_AE Envs were resistant to neutralization by BNM-III-170 and M48U1; this was expected due to the presence of the cavity-filling histidine at position 375 (H375). Interestingly, replacement of the bulky histidine by a serine at this position (H375S) did not restore neutralization sensitivity to these CD4 mimics (Fig. 2C to G). However, the H375S change in combination with the LM mutations (LM plus HS [LM+HS]) dramatically enhanced the susceptibility of both CRF01_AE strains to neutralization by both CD4 mimics. Of note, the presence of different combinations of single-layer or multiple-layer changes together with the H375S change was not sufficient to restore sensitivity to neutralization by BNM-III-170 or M48U1 (see Fig. S1C to G in the supplemental material). This was different from the results seen with respect to sCD4 neutralization, where viruses bearing LM changes without the Q61Y change were also sensitive to sCD4 neutralization (Fig. S1A and G). These data highlight subtle differences in the mode of CD4mc and sCD4 recognition of Env. Altogether, these results indicate that all of the LM changes are required to restore CD4mc sensitivity to CRF01_AE strains presenting an “empty” (H375S) Phe43 cavity.

10.1128/mBio.00280-20.2 FIG S1 Effect of single gp120 layer mutations on neutralization by CD4 and CD4mc. Recombinant HIV-1 strains expressing luciferase and bearing wild-type or mutant CRF01_AE Envs (92TH023 and CM244 isolates) were normalized by reverse transcriptase activity. Normalized amounts of viruses were incubated with serial dilutions of sCD4 (A and B), BNM-III-170 (C and D), or M48U1 (E and F) at 37°C for 1 h prior to infection of Cf2Th-CD4/CCR5 cells. Infectivity at each dilution of sCD4 or CD4mc tested is shown as the percentage of infection without sCD4 or CD4mc for each particular mutant. Quadruplicate samples were analyzed in each experiment. Data shown are the means of results obtained in at least 3 independent experiments. The error bars represent the standard deviations. Neutralization half-maximal inhibitory concentrations (IC_50_) are summarized in panel G. Download FIG S1, TIF file, 0.5 MB. Copyright © 2020 Prévost et al. 2020 Prévost et al. This content is distributed under the terms of the Creative Commons Attribution 4.0 International license.

**FIG 3:**
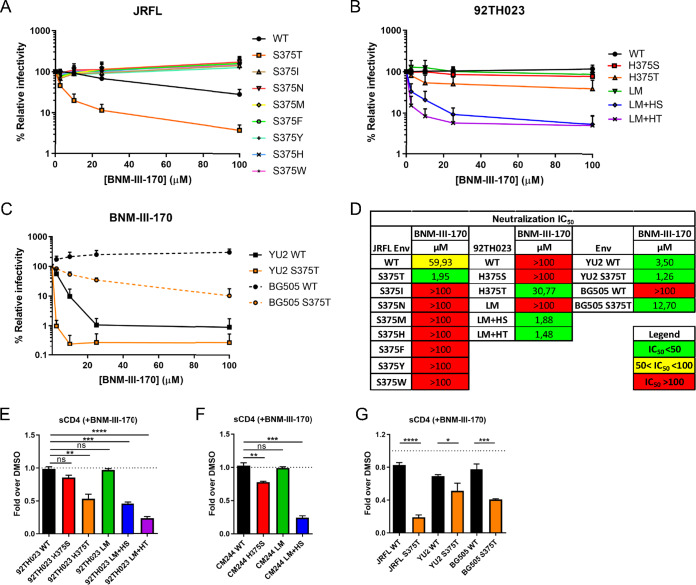
Effect of Env residue 375 on CD4mc neutralization sensitivity. (A to D) Recombinant HIV-1 strains expressing luciferase and bearing wild-type or mutant Envs (JRFL, 92TH023, YU2, and BG505 isolates) were normalized by reverse transcriptase activity. Normalized amounts of viruses were incubated with serial dilutions of BNM-III-170 (A to C) at 37°C for 1 h prior to infection of Cf2Th-CD4/CCR5 cells. Infectivity at each dilution of CD4mc tested is shown as the percentage of infection without CD4mc for each particular mutant. Quadruplicate samples were analyzed in each experiment. Data shown are the means of results obtained in at least 3 independent experiments. The error bars represent the standard deviations. Neutralization half-maximal inhibitory concentrations (IC_50_) are summarized in panel D. (E to G) Cell surface staining of 293T cells transfected with different Env expressors (92TH023, CM244, JRFL, YU2, and BG505 isolates [WT or their mutated counterparts]). Binding of sCD4 in the presence of BNM-III-170 (50 μM) or in its absence (DMSO) was detected with the anti-CD4 OKT4 MAb. Shown are the mean fluorescence intensities (MFI) obtained in the presence of BNM-III-170 normalized to the MFI in the absence of BNM-III-170 (DMSO) from the transfected (GFP^+^) population for staining obtained in at least 3 independent experiments. All MFI data were normalized to 2G12 MFI for each Env mutant. Error bars indicate means ± standard errors of the means (SEM). Statistical significance was tested using an unpaired *t* test (*, *P* < 0.05; **, *P* < 0.01; ***, *P* < 0.001; ****, *P* < 0.0001; ns, nonsignificant).

As a small residue at position 375 such as serine appears to be required for CD4mc sensitivity, we investigated the possibility that another naturally occurring small residue such as threonine (T), isoleucine (I), or asparagine (N) might affect HIV-1 sensitivity to CD4mc. We first introduced these changes into clade B HIV-1_JRFL_ Env and performed neutralization assays using the CD4mc BNM-III-170. Among all the residue 375 substitutions tested, T375 was found to be more sensitive to BNM-III-170 inhibition than the wild type (WT) (S375) and all the other variants (Fig. 3A). We then introduced this change into a CRF01_AE Env alone (H375T) and in combination with the six layer mutations (LM+HT). Strikingly, the H375T change was sufficient to sensitize the CRF01_AE strain to BNM-III-170 neutralization in the absence of the LM mutations. Thus, despite having similar small side chains, T375 was found to be sufficient to sensitize the CRF01_AE virus to neutralization by BNM-III-170 whereas S375 required the LM mutations to do so. Addition of the LM changes together with H375T (LM+HT) further increased the sensitivity of the CRF01_AE virus to BNM-III-170 neutralization, resulting in levels comparable to those seen with the LM+HS mutant (Fig. 3B and D). We confirmed the enhanced susceptibility of T375-bearing viruses to BNM-III-170 with additional clade B (YU2) and clade A1 (BG505) strains. Increased neutralization by CD4mc was seen in the presence of T375 in these two strains (Fig. 3C and D). Strikingly, HIV-1_BG505_ has intrinsic resistance to CD4mc BNM-III-170 which can be overcome by the presence of T375.

**FIG 4:**
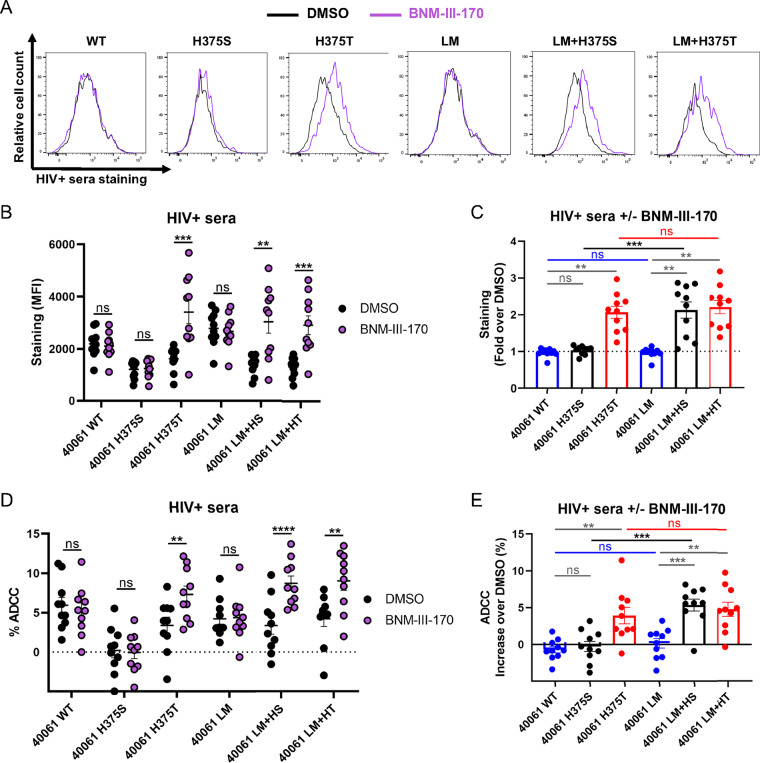
Phe43 cavity and inner domain changes enhance susceptibility of the CRF01_AE HIV-1 strain to ADCC responses mediated by HIV^+^ sera in the presence of CD4mc. (A to C) Cell surface staining of primary CD4^+^ T cells infected with a CRF01_AE transmitted-founder (TF) strain (40061) and variants with 10 different HIV^+^ sera. (A) Histograms depicting representative HIV^+^ serum staining. (B and C) The graphs shown represent the compiled mean fluorescence intensities (MFI) obtained with 10 HIV^+^ sera (B) and the fold increase in MFI in the presence of CD4mc calculated for the infected (p24^+^) population (C). (D and E) Infected primary CD4^+^ T cells were also used as target cells, and autologous PBMCs were used as effector cells in a FACS-based ADCC assay. The graphs shown represent (D) the ADCC values and (E) the increase (in percentage points) of ADCC levels obtained in the presence of CD4mc with 10 HIV^+^ sera. These results were obtained in at least 2 independent experiments. Error bars indicate means ± SEM. Statistical significance was tested using a paired *t* test or a Wilcoxon rank test based on statistical normality (**, *P* < 0.01; ***, *P* < 0.001; ****, *P* < 0.0001; ns, nonsignificant).

Since CD4mc were also shown to sensitize HIV-1-infected cells to ADCC by CD4-induced (CD4i) Abs present in HIV-positive (HIV^+^) sera (27, 33, 55–57), we evaluated the impact of the Phe43 and LM changes on the susceptibility of CRF01_AE-infected primary CD4^+^ T cells to ADCC mediated by HIV^+^ sera in the presence of the CD4mc BNM-III-170. Briefly, primary CD4^+^ T cells cultured from uninfected individuals were infected with the wild-type (WT) CRF01_AE transmitted/founder (TF) 40061 strain or with an isogenic virus with the LM and 375 changes. This infectious molecular clone (IMC) was isolated from a participant in the acute HIV-1 infection RV217 cohort (58). In line with the results presented in Fig. 2 and 3 (see also Fig. S1), replacement of CRF01_AE H375 with a threonine, but not a serine, was sufficient to gain sensitivity to CD4mc, leading to an increase in binding and ADCC mediated by HIV^+^ sera (Fig. 4). Addition of the LM changes to the resistant H375S variant conferred BNM-III-170 sensitivity but did not further enhance the susceptibility of the H375T variant.

To gain a better understanding of the impact that the LM and 375 changes have on Env conformation, we developed an assay to indirectly measure the capacity of BNM-III-170 to interact with Env. Briefly, CD4-negative cells were transfected with HIV-1_CRF01_AE_ 92TH023 or CM244 Env variants. At 48 h posttransfection, Env-expressing cells were incubated with sCD4 in the presence of the CD4mc BNM-III-170 or an equivalent amount of the vehicle (dimethyl sulfoxide [DMSO]). Binding of sCD4 to cell surface Env was detected using an anti-CD4 MAb (OKT4), which recognizes the CD4 D3 domain and therefore does not compete for gp120 binding. The ability of BNM-III-170 to interact with Env was inferred by calculating the decrease in the level of sCD4 binding to Env in the absence of CD4mc (DMSO) or in its presence (Fig. 3E to G). In agreement with neutralization results, the occupancy of the Phe43 cavity of CRF01_AE strains by BNM-III-170 was increased as a consequence of a combination of Phe43 cavity and inner domain changes (LM+HS and LM+HT) or of the H375T mutation change alone. We observed a decrease of at least 50% in sCD4 binding for both CRF01_AE strains with these mutants (Fig. 3E and F). Similarly, CD4mc competed more efficiently for sCD4 binding in the presence of the S375T change for the three additional Envs tested (JRFL, YU2, BG505) (Fig. 3G). Using this assay, we also tested a panel of anti-Env antibodies, where bNAbs (3BNC117, NIH45-46 G54W, PG16, PGT151, and PGT128) preferentially recognize the “closed” trimer (18, 19, 59) and nnAbs (17b, 19b, F240, and A32) preferentially recognize the “open” trimer (18, 19, 27, 60). In agreement with the higher level of occupancy of the Phe43 cavity by BNM-III-170 in the presence of T375 or the LM+HS mutations, for these mutants, treatment of Env-expressing cells with the CD4mc BNM-III-170 more effectively decreased binding of bNAbs to the “closed” Env conformation (Fig. S2A and B and E to G) and concomitantly enhanced binding of nnAbs to the “open” conformation (Fig. S2C and D and H to J). Overall, the nature of the residue 375 and its coevolving inner domain residues regulate the susceptibility of the CRF01_AE strains to both neutralization and ADCC.

10.1128/mBio.00280-20.3 FIG S2 Phe43 cavity and inner domain changes render the CRF01_AE HIV-1 strain susceptible to CD4mc-induced Env conformational changes. Cell surface staining of 293T cells transfected with different Env expressors (92TH023, CM244, JRFL, YU2, and BG505 isolates [WT or their mutated counterparts]) was performed using a panel of Env ligands. Binding of bNAbs (panels A and B and panels E to G) and nnAbs (panels C and D and panels H to J) was performed in the presence of BNM-III-170 (50 μM) or in its absence (DMSO). Shown are the mean fluorescence intensities (MFI) obtained in the presence of BNM-III-170 normalized to the MFI or in absence of BNM-III-170 (DMSO) from the transfected (GFP^+^) population for staining obtained in at least 3 independent experiments. All MFI data were normalized to 2G12 MFI for each Env mutant. Error bars indicate means ± SEM. Statistical significance was tested using an unpaired t-test (*, *P* < 0.05; **, *P* < 0.01; ***, *P* < 0.001; ****, *P* < 0.0001; ns, nonsignificant). Download FIG S2, TIF file, 0.5 MB. Copyright © 2020 Prévost et al. 2020 Prévost et al. This content is distributed under the terms of the Creative Commons Attribution 4.0 International license.

### Phe43 cavity and layer residues modulate the sensitivity of HIV-1 of major clades to CD4mc antiviral activities.

**FIG 5:**
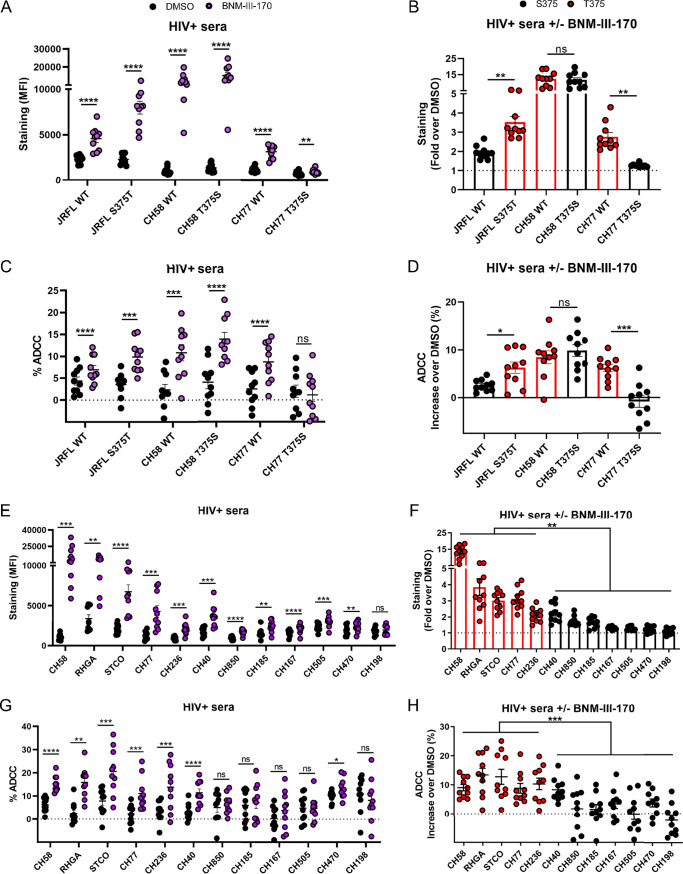
Primary viruses harboring a T375 residue are highly susceptible to ADCC responses mediated by HIV^+^ sera in the presence of CD4mc. Primary CD4^+^ T cells were infected with clade B primary HIV-1 and their variants (A to D) or with a panel of TF and chronic viruses from clades B and C (E to H). At 48 h postinfection, cell surface staining was performed with 10 different HIV^+^ sera. The graphs shown represent (A and E) the compiled mean fluorescence intensities (MFI) obtained with 10 HIV^+^ sera and (B and F) the fold increase in MFI in the presence of CD4mc calculated on the infected (p24^+^) population. Infected primary CD4^+^ T cells were also used as target cells, and autologous PBMCs were used as effector cells in a FACS-based ADCC assay. The graphs shown represent (C and G) the ADCC values and (D and H) the increases (in percentage points) in the levels of ADCC obtained in the presence of CD4mc with 10 HIV^+^ sera. These results were obtained in at least 2 independent experiments. Error bars indicate means ± SEM. Statistical significance was tested using a paired *t* test or a Wilcoxon signed-rank test (A to E and G) or an unpaired *t* test or Mann-Whitney U test (F and H), based on statistical normality (*, *P* < 0.05; **, *P* < 0.01; ***, *P* < 0.001; ****, *P* < 0.0001; ns, nonsignificant).

**FIG 6:**
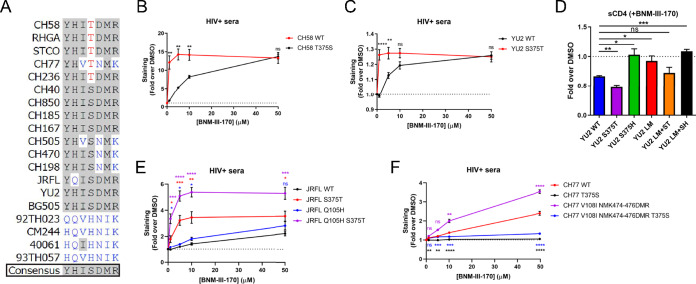
Phe43 cavity and inner domain residues modulate the susceptibility of clade B strains to CD4mc. (A) Sequence alignment of selected gp120 residues located in the Phe43 cavity (375) or the inner domain layers (61, 105, 108, 474, 475, and 476) for all HIV-1 strains used in this study. S375 and its coevolving residues are shown in black, H375 and its coevolving residues are shown in blue, and T375 is shown in red. Residues corresponding to the HIV-1 consensus sequence are shaded in gray. (B, C, E, and F) Cell surface staining with HIV^+^ sera of primary CD4^+^ T cells infected with clade B primary viruses (CH58, YU2, JRFL, and CH77) or their variants in the presence of increasing concentrations of BNM-III-170 (0, 1, 5, 10, and 50 μM). The graphs shown represent the compiled mean fluorescence intensities (MFI) obtained with 5 different HIV^+^ sera in the presence of BNM-III-170 normalized to the MFI in the absence of BNM-III-170 (DMSO) from the infected (p24^+^) population. These results were obtained in at least 2 independent experiments. (D) Cell surface staining of 293T cells transfected with an expressor of the HIV-1_YU2_ Env WT or its mutated counterparts. Binding of sCD4 in the presence of BNM-III-170 (50 μM) or in its absence (DMSO) was detected with the anti-CD4 OKT4 MAb. Shown are the mean fluorescence intensities (MFI) obtained in the presence of BNM-III-170 normalized to the MFI in the absence of BNM-III-170 (DMSO) from the transfected (GFP^+^) population for staining obtained in at least 3 independent experiments. All MFI data were normalized to 2G12 MFI for each Env mutant. Error bars indicate means ± SEM. Statistical significance was tested using an unpaired *t* test or Mann-Whitney U test based on statistical normality (*, *P* < 0.05; **, *P* < 0.01; ***, *P* < 0.001; ****, *P* < 0.0001; ns, nonsignificant).

In contrast with CRF01_AE strains, most strains from pandemic HIV-1 major clades (clades A, B, C, D, G, and CRF02_AG) harbor inner domain residues that coevolved with the presence of a small residue at position 375 (e.g., S375), making them potentially sensitive targets for CD4mc antagonist action. The presence of naturally occurring T375 in a significant fraction of these clades (up to 16.9% in clade B) might affect the susceptibility of these strains to CD4mc. Considering that T375 was found to be the residue that conferred the highest sensitivity to CD4mc in the clade B JRFL strain (Fig. 3) and among all CRF01_AE strains tested (Fig. 3 and 4), we introduced the T375 change into the HIV-1_JRFL_ IMC and evaluated the capacity of BNM-III-170 to expose epitopes recognized by sera from 10 HIV-1-infected individuals at the surface of infected primary CD4^+^ T cells. In line with the neutralization data (Fig. 3), replacement of S375 with a threonine residue (S375T) significantly enhanced the recognition of JRFL-infected cells by HIV^+^ sera in the presence of BNM-III-170 (Fig. 5A and B). We also tested two additional primary clade B viruses (HIV-1_CH58_ and HIV-1_CH77_) that naturally harbor T375 (Fig. 6A). Replacement of T375 in these two different TF viruses with a serine (T375S) strongly reduced the sensitivity of HIV-1_CH77_ to CD4mc but appeared to have no effect on HIV-1_CH58_ (Fig. 5B). However, the CH58 T375S mutant was less sensitive to CD4mc BNM-III-170 at concentrations lower than the concentration used in the staining and ADCC assays (50 μM) (Fig. 6B). A similar phenotype was also observed with another clade B strain (HIV-1_YU2_), where the variant with T375 was more sensitive to BNM-III-170 at low concentrations (Fig. 6C). In all cases, a threonine residue at position 375 enhanced Env recognition by HIV^+^ sera in the presence of BNM-III-170 (Fig. 5A and B and 6B and C). Importantly, enhanced recognition translated into enhanced susceptibility to ADCC responses (Fig. 5C and D).

Next, we evaluated a panel of 12 clade B and clade C primary viruses, including viruses from acute infections (transmitted/founder strains) (CH40, CH58, CH77, CH185, CH198, CH236, CH470, CH505, and CH850) and chronic infections (CH167, RHGA, STCO), for their susceptibility to BNM-III-170. We observed a significant increase in binding of HIV^+^ sera by BNM-III-170 with all viruses tested but one (CH198) (Fig. 5E); the increase was greater for viruses harboring a naturally occurring T375 (Fig. 5F). The higher responsiveness of viruses harboring a T375 (CH58, RHGA, STCO, CH77, and CH236) to BNM-III-170 led to a significant increase in ADCC responses for all HIV^+^ sera tested, whereas only a few (2/7) viruses harboring a S375 showed a significant increase in ADCC upon CD4mc addition (Fig. 5G and H). In brief, the presence of a naturally occurring T375 residue in clade B and clade C primary virus Envs increases susceptibility to CD4mc.

**FIG 7:**
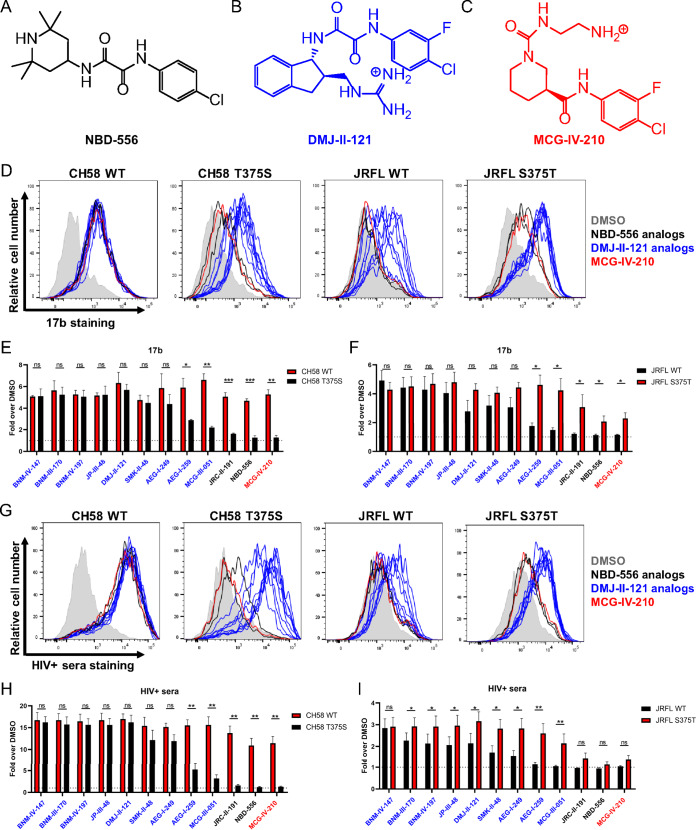
Residue 375 modulates Env sensitivity to different families of CD4mc. (A to C) Chemical structures of representative members of the different families of CD4mc tested. (D to I) Cell surface staining of primary CD4^+^ T cells infected with CH58 or JRFL viruses and their variants with anti-Env 17b MAb (D to F) or HIV^+^ sera (G to I) was performed. Shown in panels D and G are histograms depicting representative 17b and HIV^+^ serum staining results. The graphs shown represent the fold increase in mean fluorescence intensities (MFI) in the presence of CD4mc calculated on the infected (p24^+^) population obtained with 17b (E and F) or with 5 different HIV^+^ sera (H and I). These results were obtained in at least 3 independent experiments. Error bars indicate means ± SEM. Statistical significance was tested using a paired *t* test or a Wilcoxon signed-rank test based on statistical normality (*, *P* < 0.05; **, *P* < 0.01; ***, *P* < 0.001; ns, nonsignificant).

Since T375 was found to confer the highest sensitivity to BNM-III-170 in all strains tested, we sought to determine whether this was specific to this CD4mc or applicable to others as well. Therefore, we selected different generations and families of CD4mc known to engage gp120 within the Phe43 cavity. The NBD-556 compound was originally identified through a screen for Env-CD4 interaction inhibitors (61) and consists of a halogenated phenyl ring linked with a tetramethyl-piperidine via an oxalamide linker (Fig. 7A). The DMJ-II-121 compound was derived from the NBD-556 analog JRC-II-191 through the substitution of the piperidine ring for an indane ring with a guanidinium group at position 2 (Fig. 7B) to improve its Env antagonism potency (46). Other DMJ-II-121 analogs were synthesized to harbor additional chemical groups on the indane ring at position 5 (BNM-III-170, BNM-IV-147, SMK-II-48, MCG-III-051), position 6 (BNM-IV-197, JP-III-48, AEG-I-249, AEG-I-259), or position 7 (MCG-III-051) (45). To this panel of small molecules, we added a new compound (MCG-IV-210) derived from a recent high-throughput screen aimed to identify small molecules able to stabilize the CD4-bound conformation (37). Its structure resembles that of the NBD-556 analogs, but it has a shorter amide linker and an N-substituted piperidine ring (Fig. 7C). The panel of CD4mc was tested for the ability to induce Env conformational changes at the surface of primary CD4^+^ T cells infected with HIV-1_CH58_ WT or its T375S variant. Strikingly, all CD4mc tested were able to increase the exposure of the epitope recognized by the CD4i 17b MAb at the surface of WT-infected cells to similar extents (Fig. 7D to F). Introduction of the T375S change significantly diminished this ability for the less potent compounds, including MCG-IV-210, both NBD-556 analogs, and two DMJ-II-121 analogs (AEG-I-259 and MCG-III-051). The ability of these five CD4mc to enhance 17b binding to HIV-1_JRFL_-infected cells was increased by the replacement of S375 with a threonine residue (Fig. 7F). Similar results were obtained in experiments evaluating the ability of these CD4mc to increase the binding of HIV^+^ sera to CH58-infected and JRFL-infected cells (Fig. 7G to I). Altogether, our binding and functional analyses indicate that natural HIV-1 polymorphism in Ser/Thr 375 modulates Env interaction with CD4mc of different classes.

As shown in Fig. 2 to 4 (see also Fig. S1), specific inner domain layer residues (LM mutations) also modulate Env sensitivity to CD4mc in CRF01_AE strains. We investigated whether this could be extended to strains from other HIV-1 major clades. We replaced HIV-1 consensus inner domain residues in the clade B HIV-1_YU2_ Env with the residues that specifically coevolved with H375 in CRF01_AE strains (Y61H, H105Q, I108V, D474N, M475I, R476K). These changes (LM mutations) were introduced in combination with different residues at position 375 (S375, T375, or H375). Remarkably, using the sCD4 binding competition assay, the introduction of these LM changes totally abrogated the sensitivity of YU2 Env to CD4mc, but the presence of the S375T change (LM+ST) restored sensitivity to BNM-III-170 (Fig. 6D). The S375H change completely abrogated CD4mc interaction regardless of the LM mutations (Fig. 6D), as seen with CRF01_AE strains (Fig. 2, 3, and 4; see also Fig. S1). Revisiting the data shown in Fig. 5F, the three strains that were the least sensitive to BNM-III-170 were found to have inner domain residues shared with the CRF01_AE consensus sequence (Fig. 1C and 6A): CH505 (V108, N474, and K476), CH470 (N474 and K476), and CH198 (N474 and K476). Some of the LM mutations were also naturally found in two clade B strains used in this study: JRFL (Q105) and CH77 (V108, N474, and K476) (Fig. 6A). Interestingly, replacement of these residues with residues that coevolved with S375 was found to increase CD4mc sensitivity for both strains, regardless of the nature of the residue at position 375 (Fig. 6E and F). Altogether, our data show that the nature of the residue at position 375 and the coevolving gp120 inner domain layers modulate the susceptibility of HIV-1 major clades to CD4mc.

### Structural analysis of the interaction of CD4mc and HIV-1 gp120 with Phe43 cavity and inner domain layer changes.

Functional analysis clearly indicates that the LM+HT Env variant is the most effective at binding CD4mc. To better understand the structural basis for the role of the LM, HS, and HT mutations in reshaping the Phe43 cavity and in CD4mc binding, we solved the crystal structures of the CRF01_AE 93TH057 gp120 extended core (gp120_93TH057_ core_e_ [49]) containing the LM+HS changes in complex with two CD4mc compounds: MCG-IV-210 and BNM-III-170. The LM+HS gp120_93TH057_ core_e_–MCG-IV-210 complex was solved to 2.5-Å resolution (see Table S1 in the supplemental material; see also Fig. S3), enabling an assessment of the impact of the HS mutation by comparison to the LM+HT gp120_93TH057_ core_e_–MCG-IV-210 complex structure solved by us previously to 2.65-Å resolution (PDB identifier [ID] 6P9N) (37). We also solved the structure of the LM+HS gp120_93TH057_ core_e_–BNM-III-170 complex to 2.65-Å resolution (Table S1), allowing a comparison with the structure of the HS gp120_93TH057_ core_e_ (the CRF01_AE 93TH057 gp120 core_e_ with a single-point H375S mutation) in complex with DMJ-II-121, a compound closely related to BNM-III-170 (PDB ID 4I54). DMJ-II-121 lacks a (methylamino)-methyl addition to position 5 of the indane ring but is identical to BNM-III-170 in all other respects.

10.1128/mBio.00280-20.4 FIG S3 Electron density maps of CD4mc in complex with gp120. A 2Fo-Fc electron density map contoured at 1 s shows the density around MCG-IV-210 for the LM+HS gp120_93TH057_ core_e_ (left) and the LM+HT gp120_93TH057_ core_e_ (right). MCG-IV-210 is colored gray and gp120 green. Nitrogens are colored blue and oxygens red. Download FIG S3, TIF file, 2.9 MB. Copyright © 2020 Prévost et al. 2020 Prévost et al. This content is distributed under the terms of the Creative Commons Attribution 4.0 International license.

10.1128/mBio.00280-20.6 TABLE S1 X-ray crystallography data collection and refinement statistics. Download Table S1, DOCX file, 0.02 MB. Copyright © 2020 Prévost et al. 2020 Prévost et al. This content is distributed under the terms of the Creative Commons Attribution 4.0 International license.

**FIG 8:**
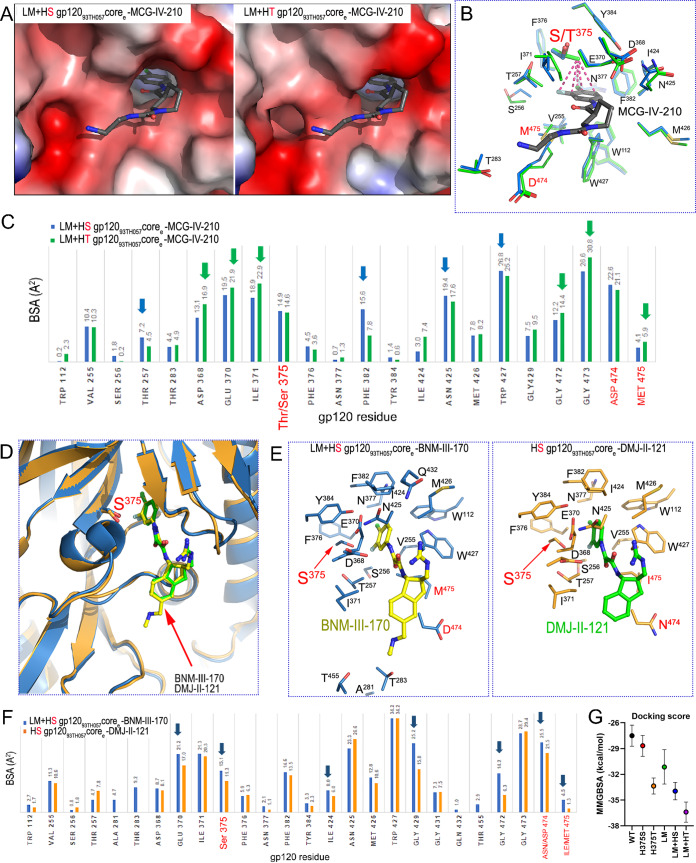
Structural effects of Phe43 cavity and inner domain changes on CD4mc docking into the Phe43 cavity. (A) Magnified views of the CD4 binding pocket of LM+HS and LM+HT gp120_93TH057_ core with MCG-IV-210 bound. Complexes are superimposed based on the gp120 core, and electrostatic surfaces are displayed over the gp120 molecule, with blue for electropositive and red for electronegative. The MGC-IV-210 compound is shown as balls and sticks. The LM+HT gp120_93TH057_ core–MCG-IV-210 complex shown is from PDB ID 6P9N. (B) Details of MCG-IV-210 interactions within the binding pocket. The side chains of gp120 residues that interact with MCG-IV-210 are shown as sticks; LM+HS is depicted in green and LM+HT in blue. MCG-IV-210 is shown with balls and sticks. MCG-IV-210 atoms that interact with the T375 γ-carbon methyl group are shown with dashes connecting to the methyl group. (C) Analysis of the MCG-IV-210 interface for binding to LM+HS and LM+HT gp120. The relative contributions of gp120 residues to compound binding are shown as buried surface area (BSA) data as calculated by PISA. BSA data represent the solvent-accessible surface area of the corresponding residue that is buried upon interface formation. (D) Structural comparison of LM+HS gp120_93TH057_ core_e_–BNM-III-170 and HS gp120_93TH057_ core_e_–DMJ-II-121 (PDB ID 4I54) complex structures. Complexes are superimposed based on the gp120 core. CD4mc compounds and Ser375 are shown as sticks. (E) Details of binding of BNM-III-170 (left panel) and DMJ-II-121 (right panel) to LM+HS gp120_93TH057_ core_e_ and HS gp120_93TH057_ core_e_, respectively. Residues forming the binding site for the CD4mc compound are shown as sticks (with Gly residues omitted) with LM residues labeled in red. (F) Analysis of the gp120 core_e_s-CD4mc binding interfaces. The relative contributions of gp120 residues to BNM-III-170 and DMJ-II-121 binding are shown by the buried surface area (BSA) as calculated by PISA. BSA represents the solvent-accessible surface area of the corresponding residue that is buried upon interface formation. (G) Docking score based on MMGBSA interaction energies, showing five replica simulations each along with averages, for six BNM-III-170-bound gp120 models.

We first sought to understand the exclusive role of residue 375, in the context of the CRF01_AE LM gp120 variant, in influencing the interaction with CD4mc. We analyzed the complex structures of LM+HS and LM+HT gp120_93TH057_ core_e_s bound to the same MCG-IV-210 compound, which displayed the highest sensitivity to position 375 changes among all CD4mc tested (Fig. 7). As shown in Fig. 8A, MCG-IV-210 bound within the hydrophobic Phe43 cavity of both gp120 mutants, anchoring deeply into the cleft. The buried surface area (BSA) measurements of the complexes were 678 Å^2^ (243 Å^2^ from gp120 and 435 Å^2^ from the compound) and 699 Å^2^ (252 Å^2^ from gp120 and 447 Å^2^ from the compound) for the complexes of LM+HS gp120_93TH057_ core_e_–MCG-IV-210 and LM+HT gp120_93TH057_ core_e_–MCG-IV-210, respectively, indicating that more surface was buried by the LM+HT variant (Fig. 8C). In addition, while the orientations of MCG-IV-210 in the binding pocket of both complexes were identical, the shapes of the pockets differed. The H375T mutation added one methyl group compared with the H375S mutation, and this addition led to the closing of the Phe43 cavity from the top. The T375 γ-carbon methyl group made a number of additional contacts with the halogenated aromatic ring of the compound, but this did not lead to an increase in the BSA at position 375 due to rearrangement of the Phe43 cavity. In fact, the presence of Thr375 led to a notable decrease in the BSA within the cavity, where residues Thr257 on the left side, Phe382 and Asn425 on the right side, and Trp427 on the bottom made more contact with the compound in the LM+HS mutant. However, residues surrounding the cavity vestibule, including Asp368, Glu370, and Ile371 at the top of the Phe43 cavity, Asn424 on the right side, and Gly472, Gly473, and Met475 at the bottom (as orientated in Fig. 8B), made more contact with MCG-IV-210 and increased the BSA in the LM+HT mutant. The increases in BSA for residues lining the Phe43 cavity may further augment the LM mutant stabilization of layer 3, which may provide an explanation for the added potency for MCG-IV-210 in the LM+HT mutant.

Next, to better understand the exclusive role of the LM residues in reshaping the Phe43 cavity for effective interaction with CD4mc, we analyzed the complex structures of BNM-III-170 and DMJ-II-121 cocrystalized with gp120_93TH057_ core_e_s that differed only in the LM residues (Fig. 8D). Structural analyses indicated that the LM+HS gp120 core_e_–BNM-III-170 complex buried more BSA and was stabilized by more contacts than the HS gp120 core_e_–DMJ-II-121 complex (Fig. 8E and F). Although some contacts observed in LM+HS gp120 core_e_–BNM-III-170 complex were not present in HS gp120 core_e_–DMJ-II-121 complex due to its smaller size [i.e., the hydrogen bond extending from the carbonyl of Gly472 to the (methylamino)methyl addition to BNM-III-170 and contacts extending from the (methylamino)methyl to Ala281, Thr283, and Thr455; Fig. 8F], there was a significant increase in the BSA of the remaining contact residues; these contacted equivalent compound atoms in the BNM-III-170 LM+HS gp120 core_e_ complex {313 Å^2^ for BNM-III-170 [296 Å^2^ when the (methylamino)methyl contribution is subtracted] versus 271 Å^2^ for DMJ-II-121}. These included contacts mediated by the highly conserved Glu370 and Ile424 residues as well as Ser375 itself, all capping the CD4mc in the Phe43 cavity from the top. There were also increased contacts to the 470-to-475 loop region (e.g., residues Gly472, Asp474, and Met475). Interestingly, two of the LM residues, Asp474 and Met475, contributed directly to CD4mc binding and increased BSA. The LM Ile475-to-Met mutation decreased the size of the Phe43 cavity and increased the hydrophobic surface available for CD4mc binding. On the other hand, Asp474 stabilized the interlayer interactions by forming a hydrogen bond to Arg476. This tightening of one side of the Phe43 binding pocket likely also led to the increase in BSA for residues on the opposite side of the pocket, including Ser375 and Gly429 (Fig. 8F). Thus, although only two of the LM residues interacted directly with BNM-III-170, their combination had an additive effect on binding and resulted in an overall increase in the complex BSA and compound affinity.

Furthermore, we conducted *in silico* analysis to predict the interaction energies present upon docking of CD4mc BNM-III-170 with our different gp120 variants. All-atom, explicit-solvent molecular dynamics (MD) simulations of BNM-III-170-bound complexes were conducted for the WT, H375S, H375T, LM, LM+HS, and LM+HT versions of gp120 core_e_. Average protein-ligand interaction energies were computed from these simulations under the generalized Born surface area (GBSA) implicit solvent model. The average relative interaction energies ranked from least to most favorable in the order WT > H375S > LM > H375T > LM+HS > LM+HT, as shown in Fig. 8G. This ordering is consistent with the ordering in half-maximal inhibitory concentration (IC_50_) values indicated in Fig. 3D, suggesting that enthalpic interactions are of high importance in determining how these residues change and how they reshape the CD4-binding site (CD4BS) to affect the activity of CD4mc.

### Phe43 cavity and inner domain substitutions shape the highly conserved CD4 binding site.

**FIG 9:**
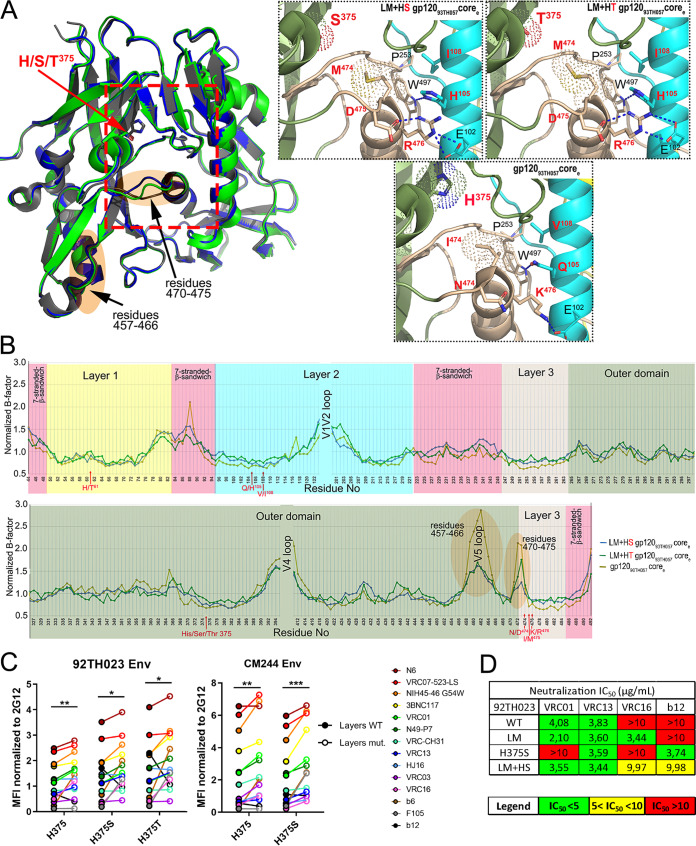
Effect of gp120 layer mutations on the highly conserved CD4 binding site. (A) Structures of LM+HS and LM+HT gp120_93TH057_ unliganded core_e_s are superimposed onto wild-type gp120_93TH057_ core_e_ (PDB code: 3TGT). Residue 375 is shown as sticks, and the gp120 regions proximal to the CD4 binding site that shows the lowest B-factors in the LM core mutants compared to the unmutated core are highlighted in orange. The magnified views (right panel) show the network of interactions in each core mediated by LM+HS/T residues. The hydrogen bonds are shown as blue dashes. LM+HS/T residues are labeled in red. (B) Plot of the normalized B-factors for the main chain atoms of residues of core_e_ structures. The rigidified region is highlighted in orange. (C) Cell surface staining of 293T cells transfected with CRF01_AE Env expressors (92TH023 and CM244 isolates [WT or their mutated counterparts]) using a panel of CD4-binding site antibodies (CD4BS Abs). Shown are the mean fluorescence intensities (MFI) normalized to 2G12 MFI obtained in the transfected (GFP^+^) population for staining obtained in at least 3 independent experiments. All MFI data were normalized to 2G12 MFI for each Env mutant. Error bars indicate means ± SEM. Statistical significance was tested using a paired *t* test or a Wilcoxon signed-rank test based on statistical normality (*, *P* < 0.05; **, *P* < 0.01; ***, *P* < 0.001). (D) Recombinant HIV-1 strains expressing luciferase and bearing wild-type or mutant CRF01_AE Envs (92TH023 and CM244 isolates) were normalized by reverse transcriptase activity. Normalized amounts of viruses were incubated with serial dilutions of VRC01, VRC16, VRC13, or b12 at 37°C for 1 h prior to infection of Cf2Th-CD4/CCR5 cells. Neutralization half-maximal inhibitory concentration (IC_50_) data are summarized.

The CD4-binding site (CD4BS) region of Env represents a highly complex quaternary arrangement of five different loops that meet to form this highly conserved structure. The following three loops converge to form the Phe43 cavity: the CD4-binding loop (residues 365 to 371), which makes critical contacts with CD4 residues F43 and R59 (1); the β20-β21 loop (residues 424 to 432), which acts as a conformational regulatory switch between the inner domain and the outer domain of gp120 (62); and the outer domain exit loop (residues 470 to 475), linking the outer domain to inner domain layer 3. Two other loops in the periphery of the Phe43 cavity were shown to be implicated in CD4 binding: loop D (residues 275 to 283) and the V5 loop (residues 457 to 468) (1). On the basis of structural analysis presented in Fig. 8, the LM+HS/T changes appear to have increased the interaction of CD4mc with the Phe43 cavity by restructuring the CD4BS, most particularly the outer domain exit loop. To better understand the molecular basis of this phenomenon, we solved crystal structures of gp120_93TH057_ core_e_s containing the LM mutations in combination with H375S (LM+HS) or H375T (LM+HT) in their unliganded state (Table S1). Unliganded structures of the LM+HS and LM+HT cores were solved at 2.2-Å and 2.5-Å resolution, respectively. The structures of LM+HS and LM+HT gp120_93TH057_ core_e_s compared to the wild-type gp120_93TH057_ core_e_ (PDB code 3TGT [49]), which carries a His at position 375, are shown in Fig. 9.

There is close similarity between the structures of the LM+HS and LM+HT core_e_s. Indeed, alignments showed that they have a root mean square deviation (RMSD) of 0.43 Å for their main chain atoms. Alignments to the wild-type gp120_93TH057_ core_e,_ resulted in RMSD values of 0.45 Å and 0.54 Å for the LM+HS and LM+HT mutants, respectively, indicating a larger dissimilarity between the LM+HT and wild-type core_e_ structures. Interestingly, a closer look into interactions formed by mutated residues in the LM+HS and LM+HT core_e_s (Fig. 9A, magnified view) reveals a new network of interactions mediated by Asp474 and Arg476 that stabilize the inner domain mobile layer 2 and 3 interface in the CD4-bound conformation (51, 63, 64). Asp474 in combination with Glu102 in layer 2 formed a network of hydrogen bonds with Arg476 in both LM mutants that bridged the two layers. This network of hydrogen bonds is absent from other clade gp120 core_e_ structures, with the exception of the clade B YU2 gp120 (PDB ID 3TGQ). In wild-type CRF01_AE gp120, Lys476 formed a hydrogen bond with Glu102, but without the added stability made possible by the Arg476 guanidinium group, leaving Asn474 out of the interface. Other LM mutant changes that potentially add to the interface stability are the Gln105-to-His mutation, which changes the character of the Trp479 amide hydrogen bond, and the Val108-to-Ile mutation, which increases the hydrophobic surface available for interaction with Pro253. CRF01_AE 93TH057 gp120 has a histidine at residue 375, in contrast to the more common Ser or Thr, which only partially fills the Phe43 cavity, leaving the space required for CD4mc compound binding. The H375S/T mutation most likely induces a loss in stability of the CD4 binding pocket in the absence of CD4 or CD4mc that in LM variants is partially compensated for by the Ile475-to-Met mutation. Both Ile475 in the wild-type gp120 and Met475 in the LM mutant pack against Trp479, which links the Phe43 pocket to the layer 3-layer 2 interface through the Trp479 amide bond to Gln105 (wild type) or His105 (LM). The bulkier side chain of Met475 provides more hydrophobic surface to the network, stabilizing and reshaping the Phe43 cavity.

The added stability of the LM mutations can be visualized by the crystallographic temperature or B-factors which describe the spread of electron density attributed to each atom (Fig. 9B). Outside the V1/V2 and V4 loop regions, which differ greatly between structures, one region formed by residues 470 to 475 stands out in the normalized B-factor plot (65). The levels of main chain atom B-factors corresponding to the residues in this region are much lower for the LM+HS and LM+HT mutants than for wild-type Env, implying that there is a greater degree of structural stability in this region in the LM mutants. It was shown previously that residues of the outer domain exit loop contribute to CD4mc binding, as also seen in the unliganded LM+HS and LM+HT core_e_s (37, 45, 48). This raises the possibility that the observed rigidification constitutes an element that reshapes the Phe43 cavity, leading to preferential binding of CD4 or CD4mc; conversely, binding of CD4 or CD4mc might stabilize layer 3, which can lead to downstream conformational changes in the gp120 inner domain. Interestingly, another region of the CD4BS was found to be significantly rigidified in the presence of the LM+HS/T mutations: the V5 loop region (residues 457 to 468). This region has previously been described as being implicated in CD4 attachment but also as being recognized by most known broadly neutralizing Abs targeting the CD4BS (e.g., VRC01). Notably, alterations in the V5 region have been associated with resistance to members of the VRC01 class of Abs (66–71) and b12 (72), consistent with a potential effect of V5 loop rigidification on gp120 recognition by CD4BS Abs. To validate our model, we evaluated the ability of a panel of CD4BS Abs to bind to CRF01_AE Envs harboring one of several different residues at position 375 (H375, S375, and T375) in combination with the LM mutations or not. Consistent with the CD4BS restructuring shown in Fig. 9A, the presence of the LM mutations was shown to increase the binding of all CD4BS Abs, except b12, regardless of the nature of the residue at position 375 in both CRF01_AE strains tested (Fig. 9C). CD4BS recognition of Env-expressing cells correlated with the neutralization profile of these mutants (Fig. 9D).

Finally, since changes in the gp120 inner domain layers in combination with changes at position 375 have a global effect on the CD4BS, we tested the sensitivity of our panel of CRF01_AE Env mutants to another class of Env antagonists targeting the CD4BS: cyclic peptide triazoles (cPTs) (73, 74). Again, the introduction of the LM mutations in combination with a small residue at position 375 (LM+HS and LM+HT) in a CRF01_AE Env was found to dramatically increase the sensitivity to neutralization by all cPTs tested (AAR029N2 and AAR029N3) (Fig. S4). However, the presence of a bulky residue at position 375 (H375) was found to abrogate this effect and conferred total resistance to the members of this class of compound (Fig. S4B to D). In summary, supported by structural data and functional assays using a wide variety of CD4BS probes, our data show that residue 375 and its inner domain coevolving residues act together to shape the highly conserved CD4BS.

10.1128/mBio.00280-20.5 FIG S4 Phe43 cavity and inner domain changes enhance the sensitivity of CRF01_AE strains to neutralization by cPTs. (A) Chemical structure of the different cPTs tested. (B to D) Recombinant HIV-1 strains expressing luciferase and bearing wild-type or mutant CRF01_AE Envs (92TH023 isolate) were normalized by reverse transcriptase activity. Normalized amounts of viruses were incubated with serial dilutions of the cyclic peptide triazoles (cPTs) AAR029N2 (B) and AAR029N3 (C) at 37°C for 1 h prior to infection of Cf2Th-CD4/CCR5 cells. Infectivity at each dilution of cPTs tested is shown as the percentage of infection without cPTs for each particular mutant. Quadruplicate samples were analyzed in each experiment. Data shown are the means of results obtained in at least 3 independent experiments. The error bars represent the standard deviations. Neutralization half-maximal inhibitory concentrations (IC_50_) are summarized in panel D. Download FIG S4, TIF file, 0.3 MB. Copyright © 2020 Prévost et al. 2020 Prévost et al. This content is distributed under the terms of the Creative Commons Attribution 4.0 International license.

## DISCUSSION

Previous studies have indicated that the gp120 inner domain plays a role in modulating the transition of Env from its unbound to its CD4-bound conformation (13–15, 75, 76). Here, we add a detailed structural understanding of this mechanism by identifying six gp120 inner domain residues able to shape the Phe43 cavity and the CD4 binding site. Collectively named layer mutants (LM), these residues determine the efficiency with which HIV-1 Envs negotiate transitions from the pretriggered state 1 conformation to CD4-bound conformations (states 2 and 3). Structures of trimeric Env stabilized in the native state 1 conformation might be required for observation of the conformational rearrangement of the gp120 inner domain layers upon CD4 or CD4mc binding. The efficiency with which a given HIV-1 variant undergoes these conformational changes influences virus susceptibility to a number of antiviral agents directed against the CD4-binding region of gp120, including CD4mc, CD4 miniproteins, cPTs, and different CD4BS antibodies. Our report documents the significant impact of the LM residues on the susceptibility of HIV-1 strain variants to this major class of virus entry inhibitors and opens the door to the rational design of agents with improved antiviral potency and breadth.

The LM residues also modulate the susceptibility of infected cells to ADCC mediated by HIV^+^ sera. Small-molecule CD4mc have been shown to enhance dramatically the ADCC-mediating potency of the antibodies present at high titers in the sera of almost all HIV-1-infected individuals (33). CD4mc are under investigation as a means to use ADCC to effect a functional “cure” of HIV-1 infection, as an adjunct to highly active antiretroviral therapy. Knowledge of the identity of the LM residues in the HIV-1 strains present in infected individuals could assist in the identification of subjects most likely to benefit from available CD4mc. The structural information on LM residues provided by our study can assist the design of CD4mc tailored to be effective against particular HIV-1 strains.

**FIG 10:**
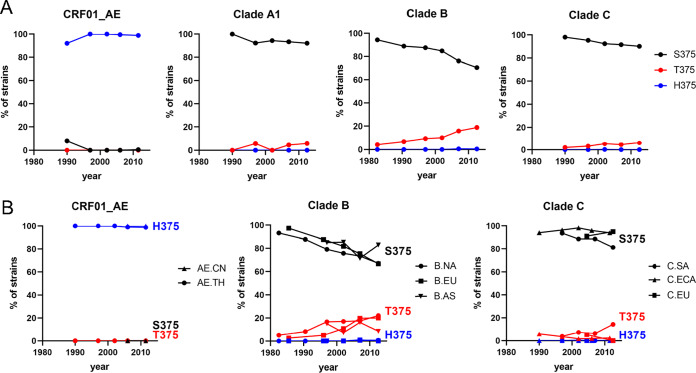
Historical changes in amino acid sequence at position 375 in diverse clades and distinct geographic regions. Env sequences from samples collected worldwide between 1979 and 2015 were examined. Totals of 335, 1,942, 1,248, and 543 Envs were used, from clades A1, B, C, and CRF01_AE, respectively. (A) Changes in frequencies of residues at position 375 in different clades. Values represent percentages of isolates from each 5-to-7-year period that contained the indicated residue. (B) Changes in frequencies of residues at position 375 in the indicated geographic regions. The residues shown in panels A and B account in most cases for more than 95% of all circulating strains. Sequences were analyzed phylogenetically to include a single Env from each patient (see Materials and Methods). For accuracy, only data from time periods with more than 25 patients are included for each clade and region. The following abbreviations are used: for clade B, NA, North America; EU, Europe; AS, Asia; for clade C, SA, Southern Africa (South Africa and Botswana); ECA, Eastern and Central Africa; IN/NP, India and Nepal; for CRF01_AE, CN, China; TH, Thailand.

Many epitopes of Env have changed at a population level during the AIDS pandemic (77). Notably, a significant decline in sensitivity of clade B circulating strains to CD4BS antibodies was observed. We examined the historical changes in residue occupancy at position 375 in diverse clades and distinct geographic regions. Among CRF01_AE strains, H375 remained highly conserved during the pandemic (Fig. 10A). In contrast, striking changes occurred in other group M major clades, most importantly in clade B, where a gradual but constant loss of S375 occurred, with S375 progressively replaced by T375 (Fig. 10A). The different patterns observed in the HIV-1 major clades and CRF01_AE, which follow similar patterns in distinct geographic regions (Fig. 10B), likely reflect differences in the constraints applied to the Phe43 cavity in the unique context of their Envs. They suggest an ongoing evolution of theCD4BS, which might need to be taken into account in developing CD4mc or immunogens designed to elicit CD4BS Abs. With respect to the issue of why T375 seems especially able to induce susceptibility to CD4mc irrespective of the LM, one clue from the structures presented here offers a possible explanation: space in the Phe43 cavity near S375 is strongly occupied by a density that may represent water or a crystallization solute, and this density is largely lacking for T375; these observations suggest higher susceptibility for T375 because it has fewer solvent molecules to eject in permitting CD4mc binding.

Taking the data together, here we show that residue 375 and its inner domain coevolving residues act together to shape the highly conserved CD4BS. This new structural understanding will assist ongoing efforts to improve CD4mc and other agents directed against the CD4BS and to design immunogens aimed at eliciting CD4BS antibodies.

## MATERIALS AND METHODS

### Ethics statement.

Written informed consent was obtained from all study participants (the Montreal Primary HIV Infection Cohort [78, 79] and the Canadian Cohort of HIV Infected Slow Progressors [80–82]), and research adhered to the ethical guidelines of CRCHUM and was reviewed and approved by the CRCHUM institutional review board (ethics committee, approval number CE 16.164–CA). Research adhered to the standards indicated by the Declaration of Helsinki. All participants were adults and provided informed written consent prior to enrollment in accordance with Institutional Review Board approval.

### Sequence analysis.

The Logo plots (83) for HIV were made using the Analyze Align tool at the HIV database and are based on the WebLogo 3 program (https://www.hiv.lanl.gov/content/sequence/ANALYZEALIGN/analyze_align.html) and the HIV-1 database global curated and filtered 2017 alignment published circa June 2018, including 1 HIV-1 Env protein sequence per person from 5,471 individuals. The relative height of each letter within individual stack represents the frequency of the indicated amino acid at that position. The numbering of all the Env amino acid sequences is based on the prototypic HXBc2 strain of HIV-1, where 1 is the initial methionine (84).

### Analysis of historical changes in amino acid sequence.

HIV-1 *env* sequences were obtained from the Los Alamos National Lab (LANL) database (https://www.hiv.lanl.gov). Nonfunctional Envs and sequences with nucleotide ambiguities were removed. Nucleotide sequences were aligned using a hidden Markov model with HMMER3 software (85), and phylogenetic relationships between sequences were inferred by the maximum-likelihood method using PhyML3 (86). A single *env* gene from each patient and a single sequence from known transmission pairs were used. In addition, a minimal distance of 0.03 nucleotide substitutions per site was applied as a cutoff for selection. Sequences were archived and residue frequencies at all Env positions in each population were determined using software developed in-house, as previously described (77).

### Cell lines and isolation of primary cells.

HEK293T human embryonic kidney cells and Cf2Th canine thymocytes (obtained from ATCC) were grown as previously described (14). Cf2Th cells stably expressing human CD4 and CCR5 (Cf2Th-CD4/CCR5) (87) were grown in medium supplemented with 0.4 mg/ml of G418 (Invitrogen) and 0.15 mg/ml of hygromycin B (Roche Diagnostics). Primary human peripheral blood mononuclear cells (PBMCs) and CD4^+^ T cells were isolated, activated, and cultured as previously described (23, 33). Briefly, PBMCs were obtained by leukapheresis and CD4^+^ T lymphocytes were purified from resting PBMCs by negative selection using immunomagnetic beads per the instructions of the manufacturer (StemCell Technologies, Vancouver, BC, Canada) and were activated with phytohemagglutinin-L (10 μg/ml) for 48 h and then maintained in RPMI 1640 complete medium supplemented with recombinant interleukin-2 (rIL-2) (100 U/ml).

### Plasmids and proviral constructs.

The plasmids expressing the CRF01_AE Envs HIV-1_92TH023_ and HIV-1_CM244_ were previously reported (41, 88). Plasmid pSVIIIenv expressing the full-length HIV-1_YU2_ Env and Tat-expressing plasmid pLTR-Tat were previously described (14). The sequence of full-length clade B HIV-1_JRFL_ (21) and clade A HIV-1_BG505_ Envs (89) were codon optimized (GenScript) and cloned into expression plasmid pcDNA3.1(-) (Invitrogen). An asparagine residue was introduced at position 332 (N332) to allow recognition of 2G12 antibody. Vesicular stomatitis virus G (VSV-G)-encoding plasmid pSVCMV-IN-VSV-G was previously described (90). For crystallographic studies, the plasmid used to express gp120 extended core (core_e_) from CRF01_AE strain 93TH057 (gp120 lacking the N and C termini and variable loops 1, 2, and 3) was previously described (49).The transmitted/founder (TF) CRF01_AE 40061 full viral genome was retrieved by single-genome amplification and cloned to generate full-length infectious molecular clone (IMC) as previously reported (91). TF and chronic IMCs of patients CH40, CH58, CH77, CH167, CH185, CH198, CH236, CH470, CH505, CH850, RHGA, and STCO were inferred, constructed, and biologically characterized as previously described (92–98). The JRFL IMC was also previously described (99).Mutations were introduced individually or in combination into the different Env expressors or IMCs using the QuikChange II XL site-directed mutagenesis protocol (Stratagene). The presence of the desired mutations was determined by automated DNA sequencing. The numbering of all the Env amino acid sequence is based on the prototypic HXBc2 strain of HIV-1, where 1 is the initial methionine (84).

### Viral production and infections.

To achieve similar levels of infection in primary CD4^+^ T cells among the different IMCs tested, VSV-G-pseudotyped HIV-1 was produced and titrated as previously described (24). Viruses were then used to infect activated primary CD4 T cells from healthy HIV-1-negative donors by spin infection at 800 × *g* for 1 h in 96-well plates at 25°C. For the viral neutralization assay, Cf2Th-CD4/CCR5 cells were infected with single-round luciferase-expressing HIV-1 (14). Briefly, 293T cells were transfected by the calcium phosphate method with the proviral vector pNL4.3(Env-)Luc and a plasmid expressing wild-type or mutant HIV-1 Env at a ratio of 2:1. Two days after transfection, the cell supernatants were harvested. The reverse transcriptase activities of all virus preparations were measured as described previously (100). Each virus preparation was frozen and stored in aliquots at –80°C until use.

### Antibodies and sera.

The following Abs were used to assess cell surface Env staining: A32, 17b, 19b, F240, PG16, N6, NIH45-46 G54W, VRC01, VRC03, HJ16, VRC-CH31, F105 (NIH AIDS Reagent Program), PGT151, PGT128 (IAVI), 3BNC117 (kindly provided by M. Nussenzweig, Rockefeller Institute, New York, NY), b6, b12 (kindly provided by D. Burton, The Scripps Research Institute, La Jolla, CA), VRC07-523-LS, VRC13, VRC16 (kindly provided by J. Mascola, Vaccine Research Center, Bethesda, MD), GE2.JG8 (kindly provided by G. Karlsson Hedestam, Karolinska Institutet, Sweden), and N49P7 (kindly provided by M. Sajadi, University of Maryland, Baltimore, MD). The anti-CD4 OKT4 monoclonal antibody (recognizing the D3 domain of CD4) (eBioscience) was used to measure the binding of sCD4 to cell surface Env. Goat anti-mouse and anti-human antibodies precoupled to Alexa Fluor 647 (Invitrogen) were used as secondary antibodies in flow cytometry experiments. Sera from HIV-infected individuals were collected, heat inactivated, and conserved at −80°C until use. A random number generator (GraphPad; QuickCalcs, San Diego, CA, USA) was used to randomly select a number of sera for each experiment.

### CD4 mimetics and other small molecules.

Soluble CD4 (sCD4) and the miniprotein M48U1 were produced and purified as previously described (14, 101). The CD4 mimetic compounds (CD4mc) NBD-556, JRC-II-191, DMJ-II-121, JP-III-48, BNM-III-170, BNM-IV-147, BNM-IV-197, MCG-IV-210, SMK-II-48, AEG-I-249, AEG-I-259, and MCG-III-051 were developed and synthesized as described previously (37, 45–47, 52, 61, 102) or as described in the “Chemical synthesis” section below. The CD4mc were analyzed, dissolved in dimethyl sulfoxide (DMSO) at a stock concentration of 10 mM, aliquoted, and stored at −80°C. Cyclic peptide triazoles (cPTs) AAR029N2 and AAR029N3 were synthesized, purified (>95% homogeneity by reverse-phase HPLC), and structurally validated as previously described (73, 74). The cPTs were analyzed, dissolved in DMSO at a stock concentration of 10 mM, aliquoted, and stored at −80°C. Each compound was then diluted to the indicated concentrations in phosphate-buffered saline (PBS) for cell surface staining, in RPMI 1640 complete medium for ADCC assays, or in complete Dulbecco’s modified Eagle’s medium (DMEM) for viral neutralization assays.

### Viral neutralization assay.

Cf2Th-CD4/CCR5 target cells were seeded at a density of 5 × 10^3^ cells/well in 96-well luminometer-compatible tissue culture plates (Perkin Elmer) 24 h before infection. Luciferase-expressing recombinant viruses (10,000 reverse transcriptase units) in a final volume of 100 μl were incubated with the indicated amounts of different proteins, compounds, or antibodies for 1h at 37°C and were then added to the target cells followed by incubation for 48 h at 37°C; the medium was then removed from each well, and the cells were lysed by the addition of 30 μl of passive lysis buffer (Promega) followed by three freeze-thaw cycles. An LB 941 TriStar luminometer (Berthold Technologies) was used to measure the luciferase activity of each well after the addition of 100 μl of luciferin buffer (15 mM MgSO_4_, 15 mM KPO_4_ [pH 7.8], 1 mM ATP, and 1 mM dithiothreitol) and 50 μl of 1 mM d-luciferin potassium salt (Prolume). The neutralization half-maximal inhibitory concentration (IC_50_) represents the amount of protein, compound, or antibody needed to inhibit 50% of the infection of Cf2Th-CD4/CCR5 cells by recombinant luciferase-expressing HIV-1 bearing the indicated Env.

### Flow cytometry analysis of cell surface staining.

Cell surface staining of infected cells was performed as previously described (24, 33). Binding of cell surface HIV-1 Env by sera (1:1,000 dilution) or anti-Env MAbs (5 μg/ml) was performed at 48 h postinfection in the presence of CD4mc or of an equal amount of the vehicle (DMSO). Infected cells were stained intracellularly for HIV-1 p24, using a Cytofix/Cytoperm fixation/permeabilization kit (BD Biosciences, Mississauga, ON, Canada) and fluorescent anti-p24 MAb (phycoerythrin [PE]-conjugated anti-p24, clone KC57; Beckman Coulter/Immunotech). The percentage of infected cells (p24^+^) was determined by gating the living cell population on the basis of viability dye staining (Aquavivid; Thermo Fisher Scientific). Samples were acquired on an LSR II cytometer (BD Biosciences), and data analysis was performed using FlowJo vX.0.7 (Tree Star, Ashland, OR, USA).

Cell surface staining of Env-expressing 293T cells was performed as previously described (103). Briefly, 2 × 10^6^ cells were transfected with 7 μg of Env expressor and 1 μg of a green fluorescent protein (GFP) expressor (pIRES-GFP) with the calcium-phosphate method. When the pSVIII Env expressor was used, it was cotransfected with 0.25 μg of a Tat-expressing plasmid. At 48 h posttransfection, 293T cells were stained with anti-Env antibodies (5 μg/ml). Alternatively, to evaluate sCD4 binding to the different cell surface Envs, transfected 293T cells were incubated with sCD4 (5 μg/ml), followed by staining performed with the monoclonal anti-CD4 OKT4 antibody (0.5 μg/ml). In experiments using transfected cells, all mean fluorescent intensities (MFI) were normalized to the MFI of 2G12 for each Env mutant.

### FACS-based ADCC assay.

Measurement of ADCC using the fluorescence-activated cell sorter (FACS)-based assay was performed at 48 h postinfection as previously described. Briefly, infected primary CD4^+^ T cells were stained with Aquavivid viability dye and cell proliferation dye (eFluor670; eBioscience) and used as target cells. Autologous PBMC effector cells, stained with another cellular marker (cell proliferation dye eFluor450; eBioscience), were added at an effector/target ratio of 10:1 in 96-well V-bottom plates (Corning, Corning, NY). A 1:1,000 final dilution of sera was added to appropriate wells, and the cells were incubated for 15 min at room temperature. The plates were subsequently centrifuged for 1 min at 300 × *g* and incubated at 37°C and 5% CO_2_ for 5 h before being fixed in a 2% PBS–formaldehyde solution. Samples were acquired on an LSR II cytometer (BD Biosciences), and data analysis was performed using FlowJo vX.0.7 (Tree Star). The percentage of ADCC resulting from gating performed on infected lived target cells was calculated with the following formula: (percentage of p24^+^ cells in targets plus effectors) − (percentage of p24^+^ cells in targets plus effectors plus sera)/(percentage of p24^+^ cells in targets).

### Protein expression and purification.

Plasmids encoding the layer mutant gp120 extended core (core_e_) proteins, HIV-1_93TH057_ gp120 core_e_ LM+HS and LM+HT, were transfected into GnT1^−^ cells using Xtremegene transfection reagent (Sigma-Aldrich) per the manufacturer’s instructions. Following 7 days of culture growth at 37°C and 8% CO_2_, cells were pelleted by centrifugation and cell supernatant was filtered. Expressed gp120 was purified by passage over a 17b affinity column made by cross-linking MAb 17b to protein A (Pierce protein A IgG plus orientation kit; Thermo Fisher). gp120 was eluted with 0.1 M glycine (pH 3.0) into tubes containing 1/10 the final volume of 1 M Tris-HCl (pH 8.5) to raise the pH. The protein was then deglycosylated with 10 units/μg of Endo H_f_ (New England Biolabs) overnight at 37°C in a mixture of deglycosylation buffer, 50 mM sodium acetate (pH 6.0), and 350 mM sodium chloride. Endo H_f_ was removed by passage over an amylose resin column, and the protein was further purified by gel filtration chromatography on a Superdex 200 16/60 column (GE Healthcare, Piscataway, NJ) equilibrated with 5 mM Tris-HCl (pH 7.2) and 150 mM sodium chloride. The protein was concentrated to approximately 5 mg/ml for use in crystallization trials.

### X-ray crystallography.

Deglycosylated HIV-1_93TH057_ gp120 core_e_ LM+HS or LM+HT (5 mg/ml) was crystalized by the hanging drop method in a mixture containing 5 to 10% polyethylene glycol (PEG) 1500, 6% PEG 400 (LM+HT), and 0.1 M HEPES (pH 7.5) or 5 to 10% PEG 3350, 6% PEG 400, and 0.1 M HEPES (pH 7.5) (LM+HS). CD4 mimetics were solubilized with DMSO at a concentration of 10 mM and diluted with crystallization buffer to 100 nM prior to use in crystal soaks. When CD4mc was added to the crystal, an equal volume of the 100 nM mimetic in crystallization buffer was added to the hanging drop and the crystals were allowed to soak for 4 h before being frozen. Crystals were flash frozen in liquid nitrogen after a brief soak in crystallization buffer containing 18% MPD (2-methyl-2,4-pentanediol) for cryoprotection and a 50 nM concentration of CD4mc when CD4mc was added.

### Data collection, structure solution, and refinement.

Data were collected on National Synchrotron Light Source II (NSLS II) highly automated macromolecular crystallography (AMX) beamline 17-ID-1 on an Eiger 9M detector system or on Stanford Synchrotron Radiation Light Source (SSRL) beamline 12-2 on a Dectris Pilatus 6M detector system. Data were integrated and processed with MOSFLM and SCALA from the CCP4 suite (104) or HKL2000 (105). Crystals were orthorhombic, belonging to space group P2_1_2_1_2_1_, with cell dimensions of a = 64.0 to 66.8 Å, b = 65.6 to 67.5 Å, and c = 86.2 to 87.8 Å, with diffraction to 2.2 to 2.65 Å. Structures were solved by molecular replacement with the program PHASER from the CCP4 suite using PDB ID 3TGT as a starting model. Model building was done using the program COOT (106). Refinement was done using the program REFMAC from the CCP4 suite or PHENIX (65).

### Structure validation and analysis.

The quality of the final refined models was monitored using the program MolProbity (107). Structural alignments were performed using the Dali server and the program LSQKAB from the CCP4 suite (104) The PISA webserver was used to determine contact surfaces and residues. All illustrations were prepared with the PyMol molecular graphic suite (DeLano Scientific, San Carlos, CA, USA). The Ramachandran plot was obtained by the use of the validation program “MolProbity” and shows that 92.8 to 97.0% of the total amino acids are in the most favored region, 3 to 6.6% in the generously allowed region, and 0 to 0.6% in the disallowed region, depending on the structure, with better values corresponding to the higher-resolution structures. Complete data collection and refinement statistics can be found in Table S1.

### *In silico* analysis.

Starting with the H375S clade A/E HIV-1 core gp120 monomer in PDB entry 4H8W (108), we generated via residue mutation the WT, H375S, H375T, LM, LM+HS, and LM+HT variants and docked BNM-III-170 to each via alignment to the 5F4P PDB entry (45). For each variant, five independent fully TIP3P-solvated MD systems were generated and run for 500 ns each using NAMD 2.13 with the CHARM36 force field, with configurations saved every 0.1 ns. For each system, molecular mechanics-generalized Born surface area (MMGBSA) interaction energy data were computed by reprocessing trajectories through NAMD and computing protein-only, ligand-only, and protein-plus-ligand potential energies under the generalized Born implicit solvent (GBIS) model. The GBIS parameters included a solvent dielectric constant of 74.69, a total molar ion concentration of 0.3, a screening cutoff of 14 Å, and surface tension of 0.0072 kcal/mol/Å^2^.

### Statistical analysis.

Statistics were analyzed using GraphPad Prism version 6.01 (GraphPad, San Diego, CA, USA). Every data set was tested for statistical normality, and this information was used to apply the appropriate (parametric or nonparametric) statistical test. *P* values of <0.05 were considered significant, and significance values are indicated as follows: *, *P* < 0.05; **, *P* < 0.01; ***, *P* < 0.001; ****, *P* < 0.0001.

### Chemical synthesis.

For details about chemical synthesis, see Text S1 in the supplemental material.

10.1128/mBio.00280-20.1 TEXT S1 Details about chemical synthesis. Download Text S1, DOCX file, 0.8 MB. Copyright © 2020 Prévost et al. 2020 Prévost et al. This content is distributed under the terms of the Creative Commons Attribution 4.0 International license.
