# Supplementary figures and images for: The HIV-1 Env gp120 Inner Domain Shapes the Phe43 Cavity and the CD4 Binding Site
Source: mBio. 2020 May 26;11(3):e00280-20. doi: 10.1128/mBio.00280-20 (PMC7251204; doi:10.1128/mBio.00280-20)

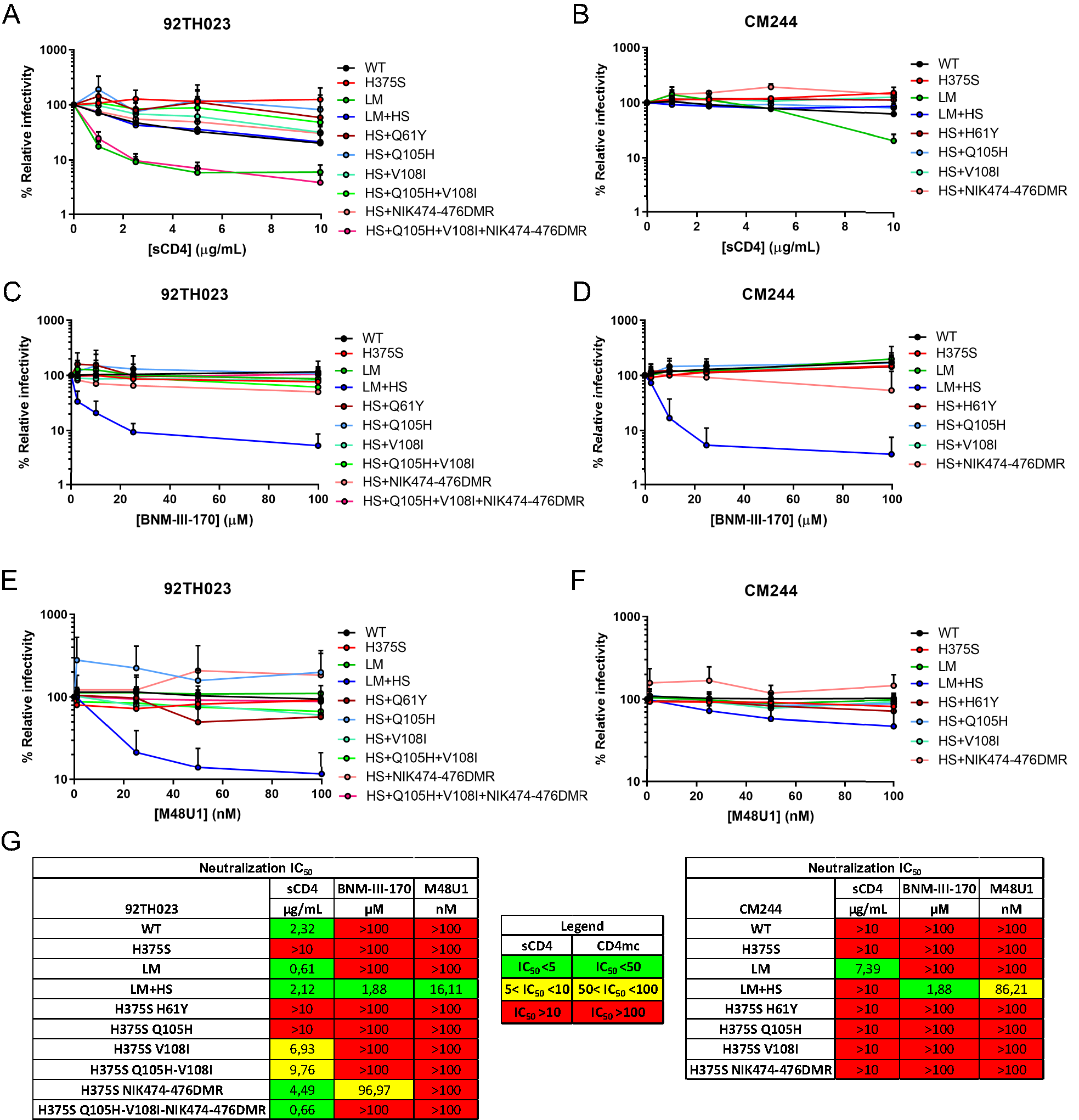

Supplement: FIG S1 [file mBio.00280-20-sf001.tif]

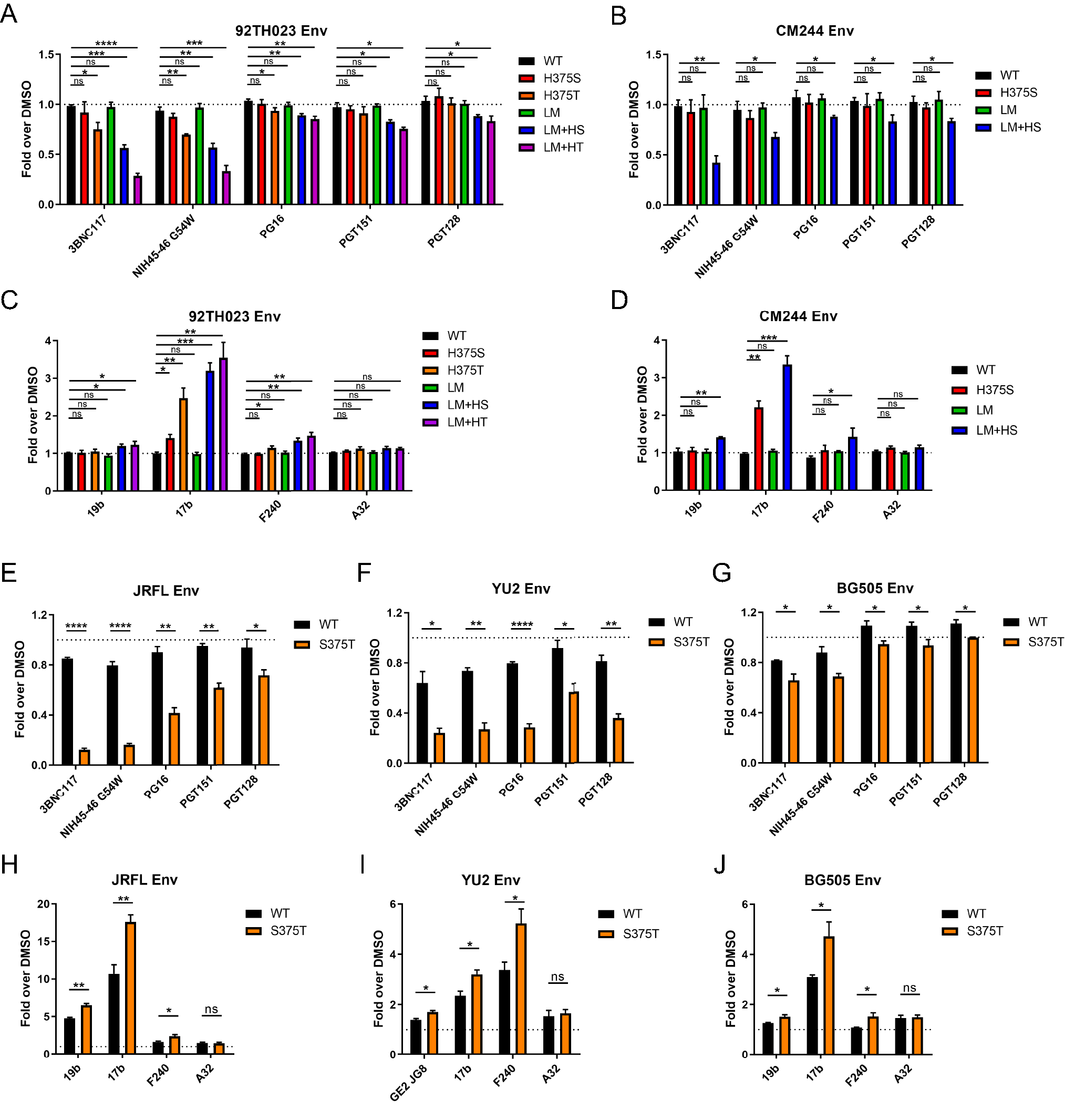

Supplement: FIG S2 [file mBio.00280-20-sf002.tif]

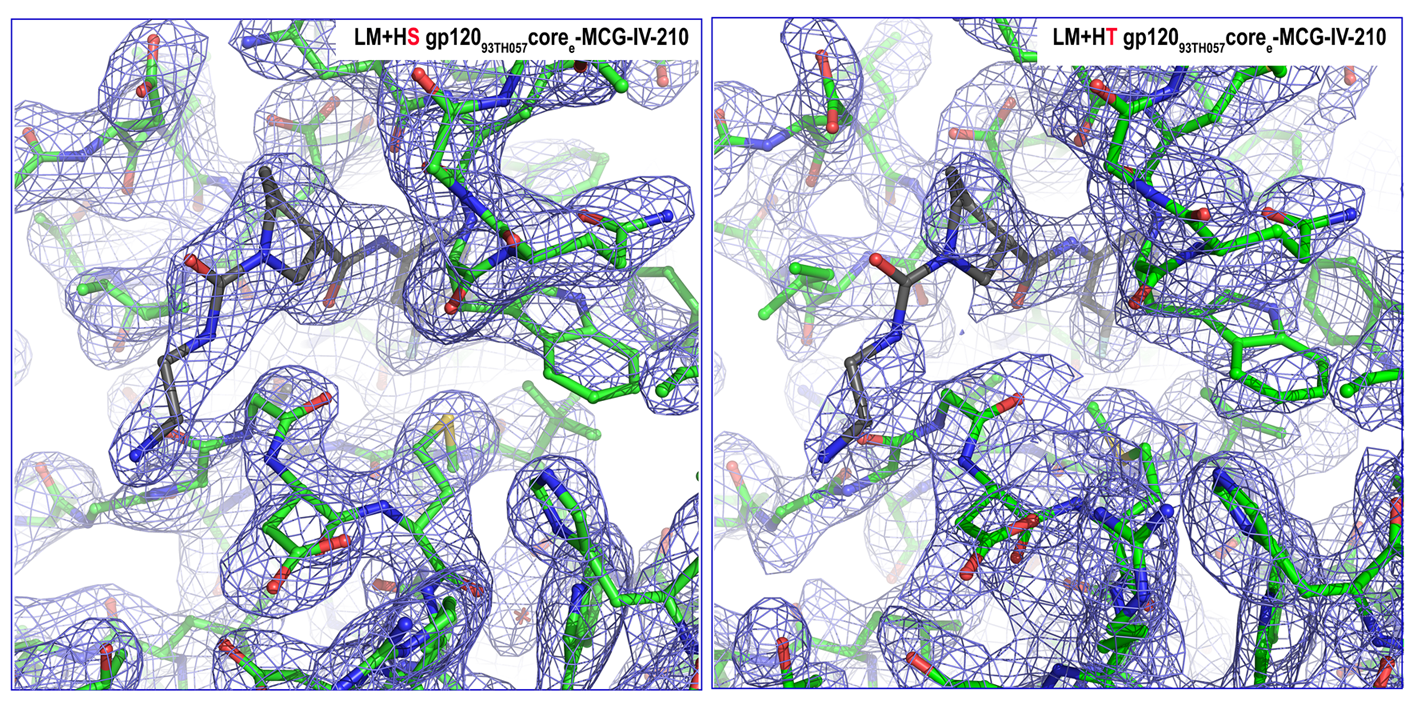

Supplement: FIG S3 [file mBio.00280-20-sf003.tif]

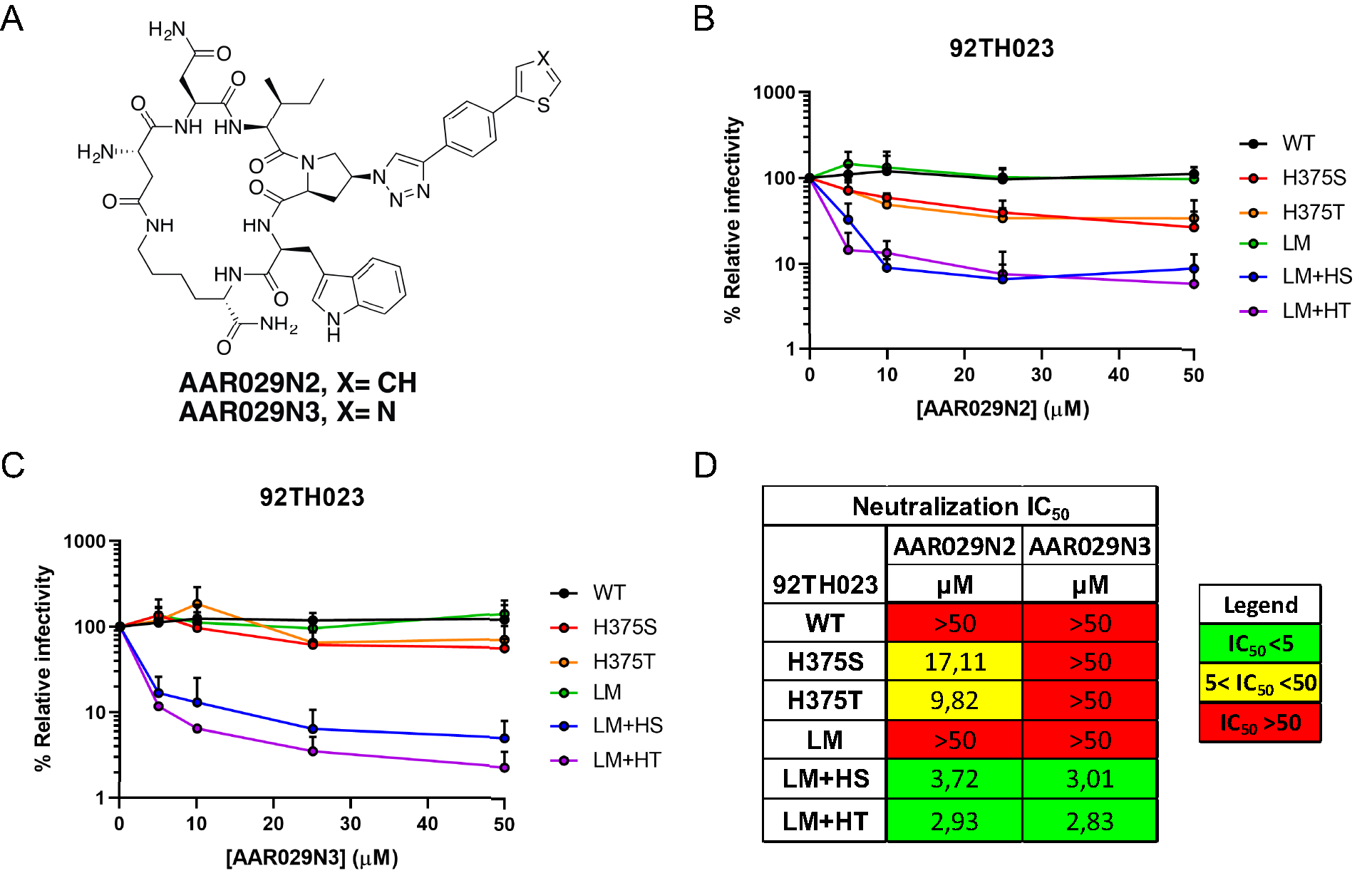

Supplement: FIG S4 [file mBio.00280-20-sf004.tif]
