# Supplementary material for: The HIV-1 Env gp120 Inner Domain Shapes the Phe43 Cavity and the CD4 Binding Site
Source: mBio. 2020 May 26;11(3):e00280-20. doi: 10.1128/mBio.00280-20 (PMC7251204; doi:10.1128/mBio.00280-20)
Supplement: TABLE S1 [file mBio.00280-20-st001.docx]

**Table S1. Data collection and refinement statistics**

|  | LM+HT gp120_CRF01_AE_ core_e_ | LM+HS gp120_CRF01_AE_ core_e_ | LM+HS gp120_CRF01_AE_ core_e_ - BNM-III-170 | LM+HS gp120_CRF01_AE_ core_e_ - (S)-MCG-IV-210 |
| --- | --- | --- | --- | --- |
| **Data collection**  Wavelength, Ǻ  Space group  Cell parameters  a, b, c,  α, β, γ, °  Complexes/a.u.  Resolution, (Å)  # of reflections  Total  Unique  R_merge_^a^, %  R_pim_^b^, %  *CC_1/2_*^c^  I/σ  Completeness, %  Redundancy | 0.920  P2_1_2_1_2_1_  66.7, 67.5, 86.8  90, 90, 90  1  50-2.5 (2.56-2.5)  55,245  13,726  16.5 (89.6)  — (—)  0.98 (0.70)  6.1 (1.0)  96.7 (62.3)  4.0 (3.2) | 1.033  P2_1_2_1_2_1_  64.0, 65.6, 87.8  90, 90, 90  1  50-2.2 (2.24-2.2)  59,449  17,485  11.3 (85.6)  6.7 (59.8)  0.99 (0.61)  14.3 (0.8)  88.0 (66.6)  3.4 (2.5) | 0.979  P2_1_2_1_2_1_  66.8, 66.5, 87.1  90, 90, 90  1  50-2.65 (2.79-2.65)  32,067  10,207  13.3 (89.9)  8.2 (57.1)  0.99 (0.49)  3.9 (0.8)  88.6 (90.0)  3.1 (3.2) | 0.979  P2_1_2_1_2_1_  65.8, 66.6, 86.2  90, 90, 90  1  50-2.5 (2.54-2.5)  38,658  10,448  11.8 (100)  6.0 (60.0)  0.97 (0.59)  14.5 (0.8)  76.5 (81.2)  3.7 (3.5) |
| **Refinement Statistics**  Resolution, Å  R^d^ %  R_free_^e^, %  # of atoms  Protein  Water  Ligand/Ion  Overall B value (Å)^2^  Protein  Water  Ligand/Ion  RMSD^f^  Bond lengths, Å  Bond angles, °  Ramachandran^g^  favored, %  allowed, %  outliers, %  PDB ID | 50.0 – 2.5  26.3  30.5  2,674  46  155  45  40  59  0.006  1.0  92.8  6.6  0.6  6UTB | 50.0 – 2.2  22.8  27.0  2,668  33  155  64  54  82  0.006  0.9  97.0  3.0  0.0  6UTD | 50.0 – 2.65  20.2  26.2  2,651  24  186  49  46  65  0.006  0.83  94.9  4.8  0.3  6UT1 | 50.0 – 2.5  24.6  30.0  2,673  3  178  61  58  67  0.007  0.95  94.9  5.1  0.0  6USW |

Values in parentheses are for highest-resolution shell

^a^*R*_merge_ = ∑│*I* - <*I*>│/∑*I*, where *I* is the observed intensity and <*I*> is the average intensity obtained from multiple observations of symmetry-related reflections after rejections

^b^R_pim_ = as defined in ([Weiss, 2001](#_ENREF_86))

^c^*CC_1/2_* = as defined by Karplus and Diederichs ([Karplus and Diederichs, 2012](#_ENREF_39))

^d^*R* = ∑║F_o_│- │ F_c_║/∑│F_o_ │, where F_o_ and F_c_ are the observed and calculated structure factors, respectively

^e^R_free_ = as defined by Brünger ([Brunger, 1997](#_ENREF_10))

^f^RMSD = Root mean square deviation

^g^Calculated with MolProbity
