# Supplementary material for: The HIV-1 Env gp120 Inner Domain Shapes the Phe43 Cavity and the CD4 Binding Site
Source: mBio. 2020 May 26;11(3):e00280-20. doi: 10.1128/mBio.00280-20 (PMC7251204; doi:10.1128/mBio.00280-20)
Supplement: TEXT S1 [file mBio.00280-20-s0001.docx]

**Chemical synthesis**

**General Considerations**

All reactions were conducted in oven-dried glassware under an inert atmosphere of nitrogen, unless otherwise stated. All solvents were reagent or high-performance liquid chromatography (HPLC) grade. Anhydrous CH_2_Cl_2_, toluene, ether and THF were obtained from the Pure SolveTM PS-400 system under an argon atmosphere. All reagents were purchased from commercially available sources and used as received. Reactions were magnetically stirred under a nitrogen atmosphere, unless otherwise noted and reactions were monitored by either thin layer chromatography (TLC) with 250 μm SiliaPlateTM pre-coated TLC plates or analytical ultraperformance liquid chromatography (UPLC). Yields refer to chromatographically or spectroscopically pure compounds. Optical rotations were measured on a JASCO P-2000 polarimeter. Proton (^1^H) and carbon (^13^C) NMR spectra were recorded on a Bruker Avance III 500-MHz spectrometer. Chemical shifts (δ) are reported in parts per million (ppm) relative to chloroform (δ 7.26), dimethyl sulfoxide (δ 2.50), acetone (δ 2.05) or methanol (δ 3.31) for ^1^H-NMR, and chloroform (δ 77.0), dimethyl sulfoxide (δ 39.4), acetone (δ 29.8) or methanol (δ 49.0) for ^13^C NMR. Infrared spectra were recorded using a JASCO 480-Plus FT-IR spectrometer, or a Perkin- Elmer Spectrum Two FT-IR spectrometer. Accurate mass measurements (AMM) were recorded at the University of Pennsylvania Mass Spectroscopy Service Center on either a Waters LCT Premier XE LC/MS or a Waters GC-TOF Premier system. Waters software calibrates and reports by use of neutral atomic masses. The mass of the electron is not included. Preparative scale HPLC was performed with a Gilson 333/334 preparative pump system equipped with a 5 mL injection loop, Sunfire C18 OBD column (10 μm packing material, 30 x 150 mm column dimensions) equipped with a UV-Vis dual wavelength (210 and 254 nm) detector and 215 liquid handling module. Solvent systems were comprised of H_2_O and acetonitrile containing 0.1% trifluoroacetic acid. Lyophilization was performed in a Labconco FreeZone 12 Plus lyophilizer (0.035 mbar). The purity of new compounds was judged by NMR and LCMS (>95%).

**Previously Reported Compounds**

The synthesis and characterization of BNM-III-170, JP-III-048, BNM-IV-147, BNM-IV-197 ([45](#bookmark)), DMJ-II-121 (46), NBD-556 ([101](#bookmark1)), JRC-II-191 ([52](#bookmark2)) and MCG-IV-210 ([37](#bookmark3)) have been previously reported.

**Synthesis of SMK-II-048**

Synthesis of aldehyde **1A** has been reported.^6^


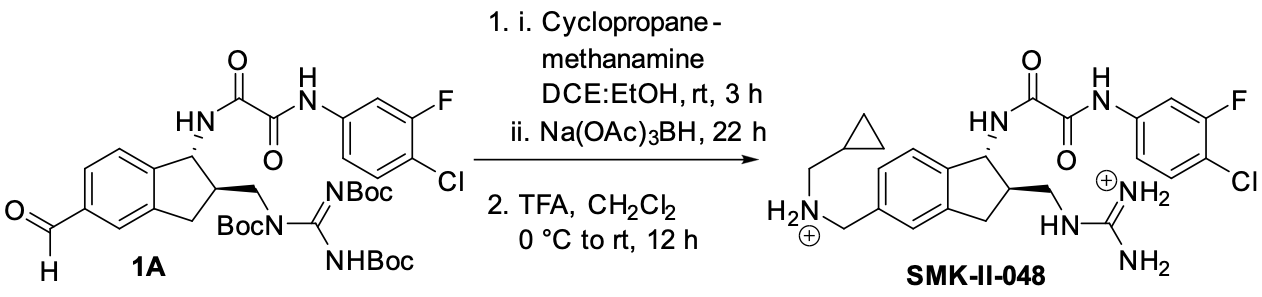


*SMK-II-048*. To a flask charged with aldehyde **1A** (30 mg, 0.041 mmol), cyclopropanemethylamine hydrochloride (44 mg, 0.41 mmol) at room temperature under nitrogen atmosphere was added dichloroethane (2 mL) and ethanol (2 mL). The reaction mixture was stirred at room temperature for 3 h before addition of sodium triacetoxyborohydride (6.0 mg, 0.103 mmol). The resulting reaction mixture was stirred at room temperature for 22 h, then cooled to 0 °C and quenched with aq. sat. NaHCO_3_ and diluted with CH_2_Cl_2_. The biphasic solution was then adjusted to pH 10 with aq. 1M NaOH and stirred for 30 min. The layers were then separated and the resulting aqueous layer was extracted with CH_2_Cl_2_. The combined organic layers were washed with aq. sat. NaCl, dried over Na_2_SO_4_, and concentrated *in vacuo*. The reside was run through a plug of silica gel, eluting with 20:80 hexanes:EtOAc containing 1% triethylamine, then concentrated *in vacuo* and used in the next step without further purification. The residue was taken up in CH_2_Cl_2_ and cooled to 0 °C before addition of trifluoroacetic acid (0.075 mL, 1.0 mmol). The solution was stirred for 12 h at room temperature, then cooled to 0 °C before addition of trifluoroacetic acid (0.05 mL, 0.6 mmol). The solution was stirred at room temperature for 24 h, then concentrated *in vacuo* and taken up in H_2_O/CH_3_CN (80:20) and subjected to HPLC purification. Conditions: eluent H_2_O/CH_3_CN (90:10 to 20:80, linear gradient); flow rate: 15 mL/min; run time: 17 min. Product-containing fractions were combined and the resulting solution was deep-frozen (-78 °C bath) and lyophilized to afford product as a white powder (3.7 mg, 12% yield from **1A**).

**1H NMR** (500 MHz, Acetone-*d*6) δ 10.33 (s, 1H), 9.72 (s, 2H), 8.87 – 8.75 (m, 2H), 7.93 (s, 1H), 7.83 (s,

2H), 7.70 (d, J = 22.1 Hz, 2H), 7.51 (t, J = 8.5 Hz, 1H), 7.46 (s, 1H), 7.36 (d, J = 8.0 Hz, 1H), 7.23 (d, J = 7.7 Hz, 1H), 5.15 (d, J = 16.1 Hz, 1H), 4.26 (s, 2H), 3.57 (d, J = 29.7 Hz, 2H), 3.29 (dd, J = 15.9, 8.3 Hz, 1H), 2.82 – 2.74 (m, 2H), 0.62 (d, J = 8.4 Hz, 2H), 0.39 (s, 2H).

1. J. Chen, J. Park, S. M. Kirk, H. C. Chen, X. Li, D. Lippincott, B. Melillo and A.B. Smith, III. *Org. Process Res. Dev.* **2019**.(doi: 10.1021/acs.oprd.9b00353)

**Synthesis of AEG-I-249 / AEG-I-259**


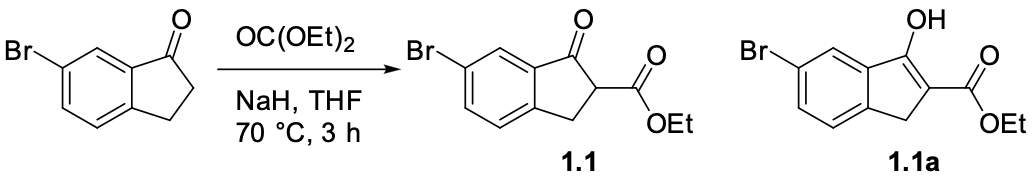


*α-ketoester* **1.1**. To a mixture of NaH (0.684 g, 17.1 mmol, 2.2 eq., 60% in mineral oil) in diethyl carbonate (5.65 mL, 46.6 mmol, 6 eq.) in a round bottom flask equipped with a reflux condenser under nitrogen gas at room temperature was added a solution of 6-bromoindanone (1.64 g, 7.77 mmol) in THF (13 mL). The reaction mixture was gradually warmed to 80 °C over 45 minutes. After another 2 hours the reaction was quenched with water. The reaction mixture was acidified to pH 2 with 1M aqueous HCl. This mixture was diluted with ethyl acetate and the layers were separated. The aqueous layer was washed with ethyl acetate (2 x 50 mL). The organic layers were combined, dried over sodium sulfate, decanted, and concentrated. Flash column chromatography (SiO_2_, 100% hexanes to 25% ethyl acetate/hexanes) afforded a red solid (2.18 g, 99%).

**^1^H NMR** (500 MHz, CDCl_3_) 1:0.5 mixture of tautomers. δ 10.31 (br. s, 0.5 H), 7.89 (s, 1 H), 7.77 (s, 1 H), 7.72 (d, *J* = 8.1 Hz, 1 H), 7.52 (d, *J* = 8.1 Hz, 0.5 H), 7.39 (d, *J* = 8.1 Hz, 1 H), 7.32 (d, *J* = 8.1 Hz, 0.5 H), 4.33 (q, *J* = 7.1 Hz, 1 H), 4.25 (q, *J* = 7.1 Hz, 2 H), 3.74 (dd, *J* = 4.0, 8.2 Hz, 1 H), 3.50 (dd, *J* = 4.0, 17.4 Hz, 2 H), 3.32 (dd, *J* = 8.3, 17.4 Hz, 1 H), 1.38 (t, *J* = 7.1 Hz, 1.5 H), 1.31 (t, *J* = 7.1 Hz, 3 H).

**^13^C NMR** (500 MHz, CDCl_3_): δ 198.2, 168.8, 152.3, 141.8, 139.1, 138.3, 137.2, 132.2, 128.2, 127.6, 126.3, 123.9, 122.1, 120.9, 104.0, 62.1, 60.5, 53.7, 32.5, 30.1, 14.6, 14.3

**IR** ν_max_ 3379, 2922, 2845, 2300, 1564, 1399, 1108, 803

**AMM** (ESI) *m/z* 281.9892 [calcd for C_12_H_11_BrO_3_ (M+H)^+^ 281.98916]


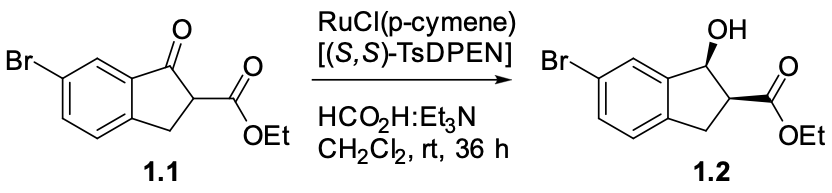


*α-hydroxyester* **1.2**. To a solution of **1.1** (1.19 g, 4.22 mol) and RuCl(*p*-cymene)[(*S,S*)-Ts-DPEN] (0.0537 g, 0.0843 mmol, 0.02 eq.) in dichloromethane (4.0 mL, 1.0 M) under nitrogen at room temperature was added 5:2 formic acid:triethylamine (1.2 mL). After three days, the reaction mixture was diluted with dichloromethane and water. The layers were separated and the aqueous layer was washed three times with dichloromethane. The organic layers were combined, dried with sodium sulfate, decanted and concentrated. The crude product mixture was purified using flash column chromatography (SiO_2_, 100% hexanes to 20% ethyl acetate/hexanes) to afford a yellow solid (1.16 g, 97%).

**^1^H NMR** (500 MHz, CDCl_3_) δ 7.55 (s, 1 H), 7.40 (d, *J* = 8.0 Hz, 1 H), 7.13 (d, *J* = 7.7 Hz, 1 H), 5.29 (d, *J* = 5.8 Hz, 1 H), 4.22 (q, *J* = 7.1 Hz, 2 H), 3.35 (m, 2H), 3.04 (dd, *J* = 7.1, 14.9 Hz, 1 H), 1.31 (t, *J* = 7.0 Hz, 3 H).

**^13^C NMR** (500 MHz, CDCl_3_): δ 172.9, 145.1, 140.7, 132.1, 128.3, 126.5, 120.8, 75.6, 61.2, 49.6, 32.7, 14.4.

**IR** ν_max_ 3409, 2917, 2843, 2362, 2350, 2335, 1365

**AMM** (ESI) *m/z* 284.0048 [calcd for C_12_H_13_BrO_3_ (M+H)^+^ 284.00481]

**[α]**_D_^23^ = - 1 ° (*c*, 2.2 CH_2_Cl_2_)


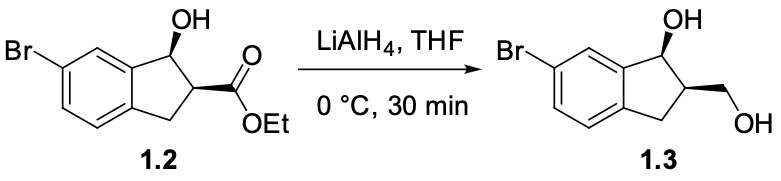


*Diol* **1.3**. To a solution of **1.2** (0.501 g, 1.76 mmol) in tetrahydrofuran (8.8 mL, 0.2 M) at 0 °C was added lithium aluminum hydride (0.100 g, 2.63 mmol, 1.5 eq.) in 5 equal aliquots in 5 minute intervals. After one hour the reaction was quenched at 0 °C with water, then saturated aqueous sodium potassium tartrate. The biphasic mixture was allowed to stir at room temperature for 30 minutes. The mixture was diluted with ethyl acetate and the layers were separated. The aqueous layer was washed three times with ethyl acetate. The organic layers were combined, dried over sodium sulfate, decanted, and concentrated. Flash column chromatography (SiO_2_, 20% ethyl acetate / hexanes to 10% methanol / ethyl acetate) to afford the desired product (0.309 g, 72%).

**^1^H NMR** (500 MHz, CDCl_3_) δ 7.55 (s, 1 H), 7.40 (dd, *J* = 1.9, 8.0 Hz, 1 H), 7.13 (d, *J* = 8.0 Hz, 1 H), 5.31 (d, *J* = 5.9 Hz, 1 H), 3.93 (m, 2 H), 2.88 (m, 2 H), 2.75 (m, 1 H).

**^13^C NMR** (500 MHz, CDCl_3_): δ 146.4, 141.8, 131.9, 128.0, 126.7, 120.5, 77.2, 63.0, 45.4, 32.5

**IR** ν_max_ 3386, 2910, 2844, 2361, 2340, 1655, 1548

**AMM** (ESI) *m/z* 241.9640 [calcd for C_10_H_11_BrO_2_ (M+H)^+^ 241.99424]

**[α]**_D_^23^ = + 41 ° (*c* 0.2, CH_2_Cl_2_)


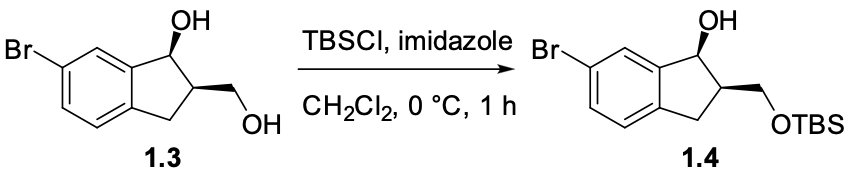


*Alcohol* **1.4**. To a solution of **1.3** (1.08 g, 4.44 mmol) in dichloromethane (44 mL, 0.1 M) under nitrogen at 0 °C was added imidazole (0.605 g, 8.88 mmol, 1.2 eq.) then TBSCl (0.803 g, 5.33 mmol, 1.2 eq.). After 1.5 hours, the reaction was quenched with water and diluted with dichloromethane. The layers were separated. The aqueous layer was washed three times with dichloromethane. The organic layers were combined, dried over sodium sulfate, decanted, and concentrated. Flash column chromatography (SiO_2_, 100% hexanes to 10% ethyl acetate / hexanes) to afford the desired product as an oil (1.45 g, 91%).

**^1^H NMR** (500 MHz, CDCl_3_) δ 7.55 (s, 1 H), 7.35 (d, 1 H, *J* = 8.0 Hz), 7.08 (d, 1 H, *J* = 8.0 Hz), 5.25 (d, 1 H, *J* = 6.5 Hz), 3.96 (dd, 1 H, *J* = 7.0, 10.4 Hz), 3.37 (br s, 1 H), 2.88 (dd, 1 H, *J* = 8.1, 16.0 Hz), 2.77 (dd, 1 H, *J* = 6.1, 16.0 Hz), 2.70 (m, 1 H), 0.87 (s, 9 H), 0.10 (s, 3 H), 0.06 (s, 3 H).

**^13^C NMR** (500 MHz, CDCl_3_): δ 147.2, 141.3, 131.3, 128.1, 126.4, 120.4, 77.1, 63.7, 45.1, 33.0, 29.8, 25.9, 18.2, -5.4, -5.5

**IR** ν_max_ 3414.35, 2955.38, 2921.63, 2850.27, 2362.37, 2335.37, 1470.46, 1254.47, 1168.65, 1089.58

**AMM** (ESI) *m/z* 355.0758 obs. C_16_H_23_BrO_2_Si [356.0807 calcd for C_16_H_25_BrO_2_Si (M+H)^+^]

**[α]**_D_^23^ = - 16 ° (*c* 0.07, CH_2_Cl_2_)


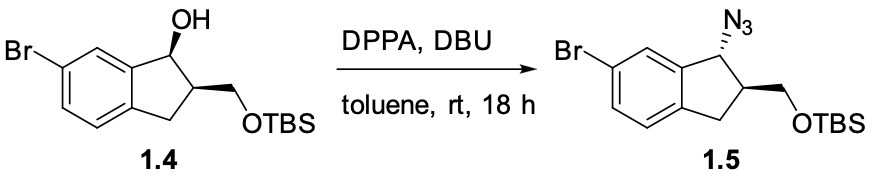


*Azide* **1.5**. To a solution of **1.4** (4.64 g, 13.0 mmol) in toluene under nitrogen at room temperature was added diphenylphosphoryl azide (5.6 mL, 26.0 mmol, 2 eq.). After ten minutes 1,8-diazabicyclo[5.4.0]undec-7-ene (5.4 mL, 36.4 mmol, 2.8 eq.) was added. The reaction mixture was then warmed to 85 °C and allowed to proceed overnight. The heating bath was removed and the reaction was quenched with water. The crude reaction mixture was diluted with water and ethyl acetate. The layers were separated and the aqueous layer was washed four times with ethyl acetate. The organic layers were combined, dried over sodium sulfate, decanted, and concentrated. Flash column chromatography (SiO_2_, 10% to 15 ethyl acetate / hexanes) to afford the desired product as an oil (4.69 g, 94%).

**^1^H NMR** (500 MHz, CDCl_3_) δ 7.51 (s, 1 H) 7.40 (d, 1 H, *J* = 8.1 Hz), 7.11 (d, 1 H, *J* = 8.4 Hz), 4.74 (d, 1 H, *J* = 6.0 Hz), 3.81 (dd, 1 H, *J* = 5.1, 10.3 Hz), 3.67 (dd, 1 H, *J* = 5.9, 10.3 Hz), 2.69 (dd, 1 H, *J* = 7.0, 15.8 Hz), 2.63 (m, 1 H), 0.91 (s, 9 H), 0.09 (overlapping s, 3 H), 0.08 (overlapping s, 3 H).

**^13^C NMR** (500 MHz, CDCl_3_): δ 143.0, 141.4, 131.8, 128.0, 126.7, 120.6, 61.7, 63.38, 49.7, 32.9, 26.0, -5.3

**IR** ν_max_ 2928, 2857, 2359, 2095, 1472, 1388, 1252, 1113, 984, 837, 777, 668

**AMM**(ESI)**:** sample did not ionize.

**[α]**_D_^23^ = - 40 ° (*c* 0.22, CH_2_Cl_2_)

^
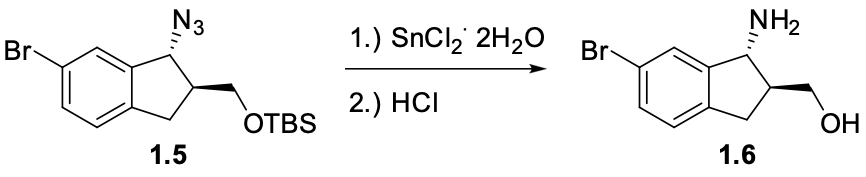
^

*Aminol* **1.6**. To a solution of **1.5** (3.32 g, 8.69 mmol) in methanol (29 mL, 0.3 M) under nitrogen at 0 °C was added tin chloride dihydrate (3.92 g, 17.4 mmol, 2 eq.). The reaction was allowed to proceed overnight. To the reaction mixture was then added 1 M aqueous HCl (15 mL). After five hours no starting material was observed. The pH of the reaction mixture was adjusted to 12 with 1 M aqueous NaOH. This solution was diluted with ethyl acetate and brine. The layers were separated. The aqueous layer was washed three times with ethyl acetate. The organic layers were combined, dried with magnesium sulfate, decanted and concentrated to afford the product (1.59 g, 75%).

**^1^H NMR** (500 MHz, DMSO): δ 7.48 (s, 1 H), 7.30 (d, 1 H, *J* = 8.0 Hz), 7.12 (d, 1 H, *J* = 7.9 Hz), 3.93 (d, 1 H, *J* = 8.5 Hz), 3.66 (dd, 1 H, 5.1, 10.6 Hz), 3.58 (dd, 1 H, *J* = 6.2, 10.6 Hz), 2.84 (dd, 1 H, *J* = 8.1, 16.0 Hz), 2.54 (partially obscured dd, 1 H, *J* = 9.5, 16.0 Hz), 2.10 (m, 1 H).

**^13^C NMR** (500 MHz, DMSO):δ 151.8, 141.5, 129.9, 127.3, 127.0, 119.7, 62.8, 59.3, 54.0.

**IR** ν_max_ 3393, 2945, 2843, 2369, 2329, 1659, 1420, 1375, 1247, 1127

**AMM**(ESI) *m/z* 242.0204 [calcd for C_10_H_12_BrNO (M+H)^+^ 242.0181]

**[α]**_D_^23^ = + 1 ° (*c* 0.18, CH_2_Cl_2_)


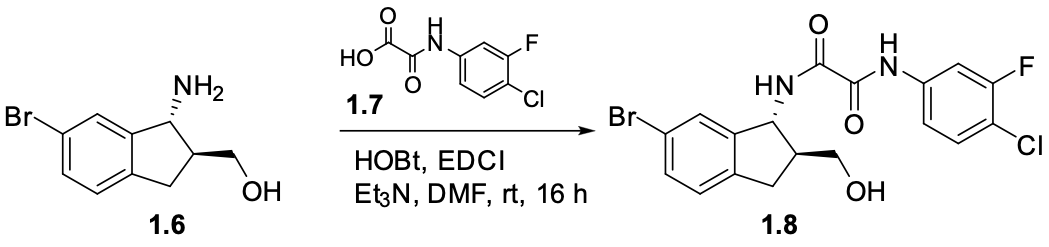


*Alcohol* **1.8**. To a solution of **1.6** (0.643 g, 2.65 mmol) and **1.7** (0.693 g, 3.18 mmol, 1.2 eq.) in dimethylformamide (6.6 mL, 0.5 M) at 0 °C under nitrogen was added EDCI hydrochloride (0.978 g, 6.36 mmol, 2.4 eq.) and hydroxybenzotriazole hydrate (1.22 g, 6.36 mmol, 2.4 eq.). After five minutes triethylamine was added (1.3 mL, 6.36 mmol, 2.4 eq.) and the reaction was allowed to proceed overnight. The reaction mixture was quenched with saturated aqueous sodium bicarbonate. The reaction mixture was diluted with water and ethyl acetate. The layers were separated and the aqueous layer was washed with ethyl acetate. The aqueous layer was adjusted to pH 9 with 1 M aqueous sodium hydroxide and washed twice with ethyl acetate. The organic layers were combined, dried with magnesium sulfate, and decanted. The organic layer was loaded onto celite and the product was purified with a silica gel plug (50% ethyl acetate / hexanes to 10% methanol / ethyl acetate) to afford the product as a white solid (0.763 g, 65%).

**^1^H NMR** (500 MHz, DMSO): δ 11.06 (s, 1 H), 9.42 (d, 1 H, *J* = 8.7 Hz), 7.97 (dd, 1 H, *J* =2.3, 11.8 Hz), 7.75 (app. d, 1 H, *J* = 8.8 Hz), 7.60 (t, 1 H, *J* = 8.7 Hz), 7.39 (app. d, 1 H, *J* = 7.6 Hz), 7.32 (s, 1 H), 7.21 (d, 1 H, *J* = 8.0 Hz), 5.22 (t, 1 H, *J* = 8.4 Hz), 4.75 (t, 1 H, *J* = 5.0 Hz), 3.54 (m, 2 H), 3.00 (m, 1 H), 2.70 (m, 2 H).

**^13^C NMR** (500 MHz, DMSO): δ 160.6, 159.5, 157.4 (d, *J*_CF_ = 244 Hz), 156.4, 146.5, 142.3, 139.0 (d, *J*_CF_ = 10 Hz), 131.2, 130.9, 127.4, 127.2, 119.7, 118.0 (d, *J*_CF_ = 3 Hz), 114.9 (d, *J*_CF_ = 17 Hz), 109.1 (d, *J*_CF_ = 26 Hz), 62.1, 56.5, 49.1, 33.5.

**IR** ν_max_ 3372, 3258, 2919, 2367, 1660, 1515, 1060, 956, 674, 441, 426

**AMM**(ESI) *m/z* 441.0021 [calcd for C_18_H_15_BrClFN_2_O_3_ (M+H)^+^ 441.0017]

**[α]**_D_^23^ = + 16 ° (*c* 0.26, CH_2_Cl_2_)


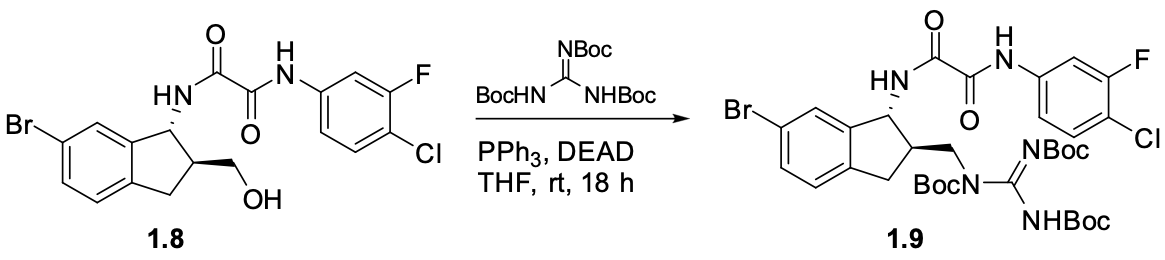


*Bromide* **1.9**. To a solution of alcohol **1.8** (0.180 g, 0.407 mmol) and triphenylphosphine (0.427 g, 1.63 mmol, 4 eq.) in tetrahydrofuran (15 mL, 0.02 M) under nitrogen at room temperature was added tribocguanidine (0.242 g, 0.403 mmol, 0.99 eq.). To this mixture was slowly added diethyl azodicarboxylate (0.43 mL, 1.63 mmol, 4 eq.) and the reaction was allowed to proceed overnight. The reaction was quenched with aqueous sodium bicarbonate and diluted with ethyl acetate and water. The layers were separated and the aqueous layer was washed three times with ethyl acetate. The organic layers were combined, dried with sodium sulfate, decanted and concentrated. The crude reaction mixture was purified using flash column chromatography (SiO_2_, dry loading on celite, 10% ethyl acetate / hexanes) to afford a white solid (0.108 g, 68%).

**^1^H NMR** (500 MHz, CDCl_3_): δ 10.61 (br s, 1 H), 9.38 (s, 1 H), 7.89 (d, 1 H, *J* = 9.0 Hz), 7.76 (dd, 1 H, *J* = 2.4, 10.6 Hz), 7.37 (m, 2 H), 7.33 (s, 1 H), 7.27 (m, 1 H), 7.11 (d, 1 H, *J* = 8.7 Hz), 5.24 (t, 1 H, *J* = 8.1 Hz), 4.13 (m, 2 H), 3.14 (dd, 1 H, *J* = 7.9, 15.8 Hz), 2.91 (m, 1 H), 2.74 (dd, 1 H, *J* = 9.0, 15.8 Hz), 1.51 (m, 27 H).

**^13^C NMR** (500 MHz, CDCl_3_): δ 159.74, 158.2 (d, *J*_CF_ = 248 Hz), 157.32, 153.39, 153.25, 143.64, 140.81, 136.5 (d, *J*_CF_ = 9.6 Hz), 131.52, 130.94, 127.20, 126.55, 120.70, 117.2 (d, *J*_CF_ = 18.1 Hz), 116.04 (d, *J*_CF_ = 3.5 Hz), 108.59 (d, *J*_CF_ = 26.1 Hz), 83.79, 58.44, 49.78, 48.12, 34.72, 29.84, 28.38, 28.19, 28.16, 28.13, 28.09, 27.96.

**IR** ν_max_ 3277, 2972, 2927, 2852, 2360, 1762, 1664, 1610, 1517, 1251, 1148, 664

**AMM**(ESI) *m/z* 782.1984 [calcd for C_34_H_42_BrClFN_5_O_8_ (M+H)^+^ 782.1968]

**[α]**_D_^23^ = + 15 ° (*c* 0.4, CH_2_Cl_2_)


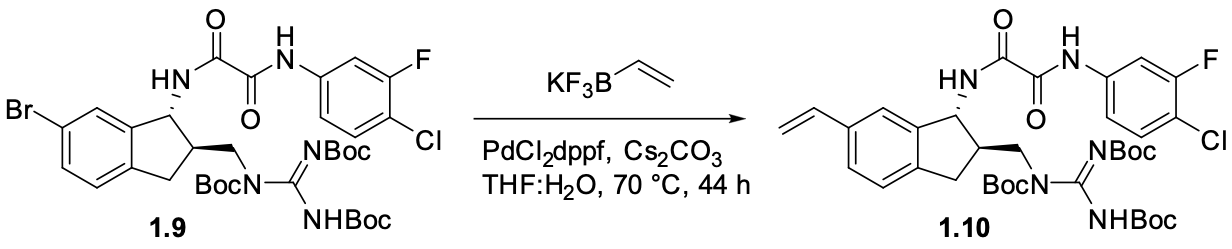


*Alkene* **1.10**. Bromide **1.9** (0.0987 g, 0.126 mmol), Pd(dppf)Cl_2_ (0.0082 g, 0.0101 mmol, 0.08 eq.), vinyltrifluoroborate (0.0506 g, 0.378 mmol, 3 eq.) and cesium carbonate (0.164 g, 0.504 mmol, 4 eq.) under nitrogen at room temperature were dissolved in nitrogen-sparged THF/water (32 mL, 10:1, 0.004 M) in a round bottom flask equipped with a reflux condenser. The reaction mixture was heated to reflux overnight. After 21 h, the reaction mixture was cooled to room temperature and diluted with water. The reaction mixture was then filtered through celite with diethyl ether and diluted with water. The layers were separated and the aqueous layer was washed three times with diethyl ether. The organic layers were combined, dried over sodium sulfate, decanted and concentrated. The crude product mixture was purified using flash column chromatography (SiO_2_, 20% hexanes / ethyl acetate) to afford product as a white solid (0.058 g, 63%).

**^1^H NMR** (500 MHz, CDCl_3_): δ10.55 (br s, 1 H), 9.38 (s, 1 H), 7.82 (d, 1 H, *J* = 9.1 Hz), 7.76 (dd, 1 H, *J* = 2.5 , 10.6 Hz), 7.38 (t, 1 H, *J* = 8.3 Hz), 7.30 (m, 1 H), 7.25 (m, 1 H), 7.23 (s, 1H), 7.19 (d, 1 H, *J* = 7.8 Hz), 6.67 (dd, 1 H, *J* = 10.9, 17.5 Hz), 5.69 (app. d, 1 H, *J* = 17.6 Hz), 5.25 (t, 1 H, *J* = 8.7 Hz), 5.20 (d, 1 H, *J* = 10.9 Hz), 4.14 (m, 1 H), 3.17 (dd, 1 H, *J* = 7.8, 15.8 Hz), 2.88 (m, 1 H), 2.79 (dd, 1 H, *J* = 8.8, 15.7 Hz), 1.56 - 1.44 (m, 27 H).

**^13^C NMR** (500 MHz, CDCl_3_):δ 159.7, 158.2 (d, *J*_CF_ = 248 Hz), 157.5, 153.3, 153.2, 141.7 (d, *J*_CF_ = 8 Hz), 137.0, 136.6, 136.5, 130.9, 126.8, 125.1, 121.5, 117.1 (d, *J*_CF_ = 18 Hz), 116.0 (d, *J*_CF_ = 3 Hz), 113.8, 108.4 (d, *J*_CF_ = 26 Hz), 83.7, 58.5, 50.0, 48.4, 35.0, 28.2, 28.2, 28.1

**IR** ν_max_ 3277, 2977. 2924, 2857, 2360, 2336, 1757, 1664, 1517, 1366, 1243, 1150 668

**AMM**(ESI) *m/z* 752.2864 [calcd for C_36_H_45_ClFN_5_O_8_ (M+Na)^+^ 752.2838]

**[α]**_D_^23^ = + 19 ° (*c* 0.5, CH_2_Cl_2_)


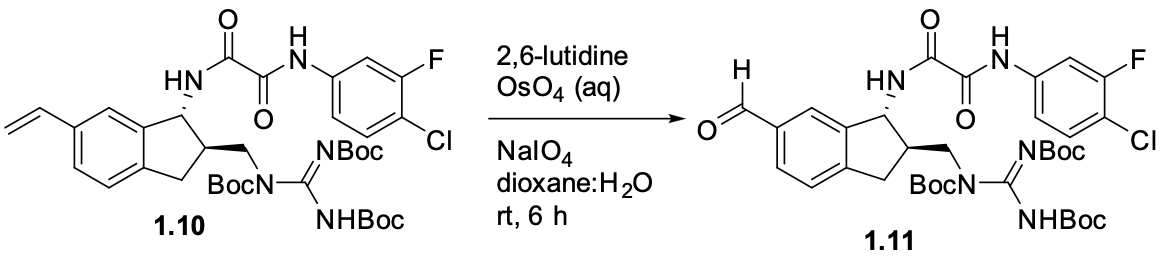


*Aldehyde* **1.11**. To a solution of alkene **1.10** (0.136 g, 0.186 mmol) in THF/water (3:1, 3 mL, 0.05 M) was added osmium tetroxide (2 mol% in water, 0.95 mL, 0.019 mmol, 0.1 eq.) then sodium periodate (0.120 g, 0.559 mmol, 3 eq). After three hours, the reaction was quenched with saturated aqueous sodium thiosulfate and allowed to stir overnight. The reaction mixture was diluted with ethyl acetate and the layers were separated. The aqueous layer was washed three times with ethyl acetate. The organic layers were combined, dried with magnesium sulfate, decanted and concentrated. The crude product mixture was purified using flash column chromatography (SiO_2_, 100% hexanes to 30% ethyl acetate / hexanes, ethyl acetate flush) to afford the product as a solid (0.112 g, 82%).

**^1^H NMR** (500 MHz, CDCl_3_): δ 10.59 (br s, 1 H), 9.96 (s, 1 H), 9.31 (s, 1 H), 7.90 (d, 1 H, *J* = 8.7 Hz), 7.79 (d, 1 H, *J* = 7.7 Hz), 7.75 (m, 1 H), 7.72 (s, 1 H), 7.39 (t, 2 H, *J* = 8.3 Hz), 7.24 (d, 1 H, *J* = 8.6 Hz), 5.31 (t, 1 H, *J* = 8.7 Hz), 4.16 (m, 1 H), 3.27 (dd, 1 H, *J* = 7.5, 16.1 Hz), 2.92 (m, 2 H), 1.59 – 1.43 (m, 27 H).

**^13^C NMR** (500 MHz, CDCl_3_): δ 191.7, 159.8, 158.3 (d, *J*_CF_ = 248 Hz), 157.3, 153.6, 153.2, 149.2, 142.7, 136.4 (d, *J*_CF_ = 10 Hz), 136.1, 131.0, 130.7, 125.7, 125.1, 117.2 (d, *J*_CF_ = 18 Hz), 116.0 (d, *J*_CF_ = 4 Hz), 108.5 (d, *J*_CF_ = 26 Hz), 83.8, 58.3, 49.8, 48.0, 35.5, 28.2, 28.12, 28.08

**IR** ν_max_ 3393, 3284, 2979, 2932, 2852,1792, 1725, 1666, 1606, 1517, 1369, 1295, 1248, 1147, 775

**AMM**(ESI) *m/z* 732.2798 [calcd for C_35_H_43_ClFN_5_O_9_ (M+H)^+^ 732.2812]

**[α]**_D_^23^ = + 31 ° (*c* 2.0, CH_2_Cl_2_)


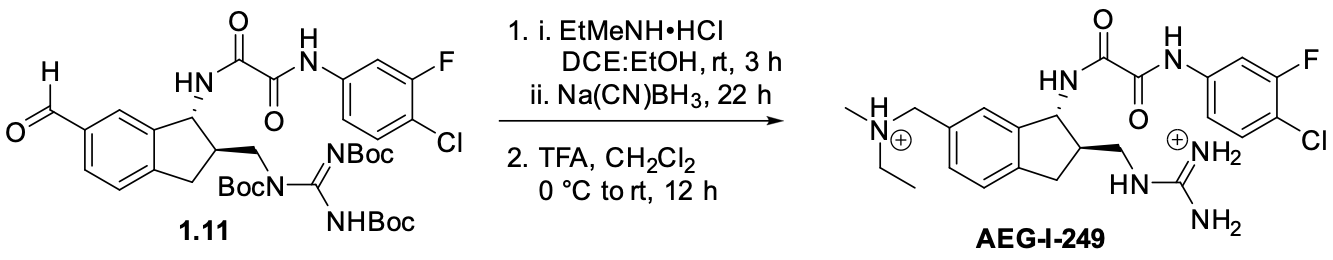


**AEG-I-249**. To a solution of **1.11** (0.030 g, 0.039 mmol) in dichloroethane (3 mL, 0.02 M) with 3 Å molecular sieves under nitrogen atmosphere at room temperature was added ethylmethylamine (0.08 mL, 0.948 mmol, 20 eq.). After stirring for one hour, sodium triacetoxyborohydride (0.016 g, 0.26 mmol, 5 eq.) was added. After 7 hours, no starting material was observed by TLC or LC/MS. The reaction was quenched with saturated aqueous sodium bicarbonate and diluted with ethyl acetate. The layers were separated, and the aqueous layer was washed three times with ethyl acetate. The organic layers were combined, dried over sodium sulfate, decanted, and concentrated. Flash column chromatography (SiO_2_, 0% to 5% MeOH in dichloromethane) afforded a partially purified amine. This amine was dissolved in dichloromethane (3.9 mL, 0.01 M). To this solution was added trifluoroacetic acid (0.12 mL, 1.6 mmol, 40 eq.). After two days complete conversion was observed by LC/MS. The reaction mixture was concentrated, dissolved in 2.3 mL 1:1 acetonitrile/water and purified by reverse phase HPLC (15 mL/min, 15-70% H_2_O/ACN + 0.1% TFA, 10 min gradient). The isolated fractions were frozen and concentrated by lyophilization to afford **AEG-I-249** as a white solid (10 mg, 37% over two steps).

**^1^H NMR** (500 MHz, acetone-d6): δ 11.79 (s, 1 H), 10.30 (s, 1 H), 8.80 (m, 2 H), 7.99 (ddd, 1 H, *J* = 1.0, 2.4, 11.6 Hz), 7.90 – 7.65 (br s, 2 H), 7.72 (m, 1 H), 7.55 – 7.46 (m, 3 H), 7.33 (d, 1 H, *J* = 7.7 Hz), 5.34 (t, 1 H, *J* = 8.4 Hz), 4.55 – 4.23 (br d, *J* = 80.9 Hz), 3.68 – 3.55 (m, 2 H), 3.41 – 3.24 (br s, 1 H), 3.30 (dd, 1 H, *J* = 8.0, 15.9 Hz) 3.21 – 3.08 (br s, 1 H), 2.96 (m, 1 H), 2.84 (dd, 1 H, *J* = 9.0, 15.9 Hz), 2.78 (s, 3 H), 1.37 (t, 3 H, *J* = 7.2 Hz)

**^13^C NMR** (500 MHz, acetone-d6, mixture of rotamers): δ 161.7 (q, *J_CF_* = 34.0 Hz, TFA), 161.0609, 160.0 (d, *J_CF_* = 249 Hz), 158.6 (d, *J_CF_* = 245 Hz), 159.3, 159.2, 159.2, 159.1, 159.1, 144.5, 143.8, 139.2 (d, *J_CF_* = 10.1 Hz), 139.1 (d, *J_CF_* = 10.0 Hz), 132.0, 131.6, 129.8, 127.7, 126.3, 117.9 (q, *J_CF_* = 294 Hz), 117.9 (d, *J_CF_* = 3.5 Hz), 117.1 (d, *J_CF_* = 3.5 Hz), 116.3 (d, *J_CF_* = 18.0 Hz), 109.3 (d, *J_CF_* = 26.2 Hz), 109.2 (d, *J_CF_* = 26.1 Hz), 59.4, 58.8, 58.7, 51.2, 51.0, 48.0, 48.0, 44.6, 44.5, 38.7, 35.1, 35.1, 9.4.

**IR** ν_max_ 3355, 2359, 2341, 1682, 1517, 1428, 1203, 1135, 975, 837, 801, 722, 668

**AMM**(ESI) *m/z* 475.2011 [calcd for C_23_H_29_ClFN_6_O_2_ (M+H)^+^ 475.2025]

**[α]**_D_^23^ = + 9 ° (*c* 0.09, MeOH)


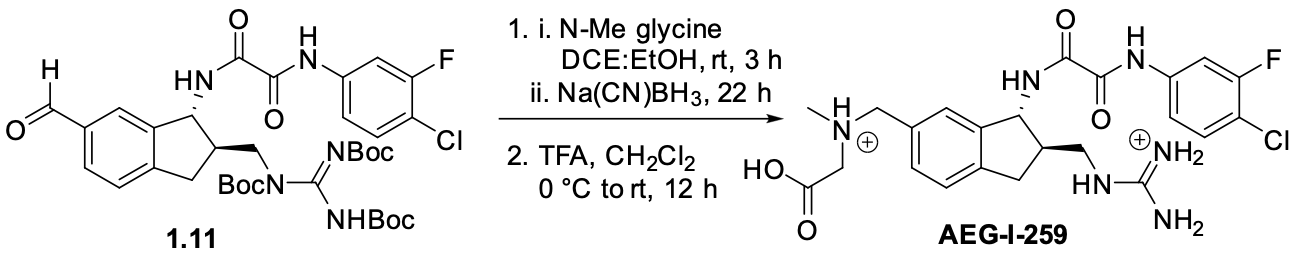


**AEG-I-259**. To a solution of **1.11** (0.060 g, 0.079 mmol) in dichloroethane (2 mL, 0.04 M) with 3 Å molecular sieves under nitrogen atmosphere at room temperature was added a solution of sarcosine (0.140 g, 1.57 mmol, 20 eq.) in methanol (2 mL). After stirring for 30 minutes, sodium triacetoxyborohydride (0.083 g, 0.39 mmol, 5 eq.) was added. After 24 hours, no conversion was observed by LC/MS. Sodium cyanoborohydride (0.030 g, 0.48 mmol, 6 eq.) was then added. After two hours, no starting material was observed by TLC or LC/MS. The reaction was quenched with saturated aqueous sodium bicarbonate and diluted with ethyl acetate. The layers were separated, and the aqueous layer was washed three times with ethyl acetate. The organic layers were combined, dried over sodium sulfate, decanted, and concentrated. Flash column chromatography (SiO_2_, 0% to 10% MeOH in dichloromethane) afforded a partially purified amine. This amine was dissolved in dichloromethane (5.5 mL, 0.01 M). To this solution was added trifluoroacetic acid (0.16 mL, 2.2 mmol, 40 eq.). After two days complete conversion was observed by LC/MS. The reaction mixture was concentrated, dissolved in 2.3 mL 1:1 acetonitrile/water and purified by reverse phase HPLC (15 mL/min, 5-80% H_2_O/ACN + 0.1% TFA, 20 min gradient). The isolated fractions were frozen and concentrated by lyophilization to afford **AEG-I-259** as a white solid (6.8 mg, 12% over two steps).

**^1^H NMR** (500 MHz, acetone-d6): δ 11.10 (s, 1 H), 9.47 (d, 1 H, *J* = 9.1 Hz), 7.98 (dd, 1 H, *J* = 2.4, 11.8 Hz), 7.83 (m, 1 H), 7.77 (dd, 1 H, *J* = 2.3, 8.9 Hz), 7.60 (t, 1 H, *J* = 8.7 Hz), 7.31 (s, 1 H), 7.25 (s, 1 H), 5.20 (t, 1 H, *J* = 8.8 Hz), 4.09 (s, 2 H), 3.66 (s, 2 H), 3.39 (m, 1 H), 3.12 (dd, 1 H, *J* = 8.0, 15.8 Hz), 2.83 (m, 1 H), 2.68 (dd, 1 H, *J* = 9.3, 15.7 Hz), 2.58 (s, 3 H).

**^13^C NMR** (500 MHz, acetone-d6): δ 168.5, 159.9, 158.8, 156.8 (q, *J_CF_* = 34.0 Hz, TFA), 157.1, 156.8 (d, *J_CF_* = 244 Hz),143.0, 142.9, 142.1,138.3 (d, *J_CF_* = 10.0 Hz),130.7, 130.2, 125.6, 124.9, 117.3 (d, *J_CF_* = 10.0 Hz), 117.2 (q, *J_CF_* = 299.7 Hz, TFA), 114.4 (d, *J_CF_* = 18.0 Hz), 108.5 (d, *J_CF_* = 25.8 Hz), 59.1, 56.9, 55.2, 45.6, 42.9, 40.5, 33.7.

**IR** ν_max_ 3398, 2917, 2849 2359, 2342, 1683, 1672, 1645, 1635, 1626, 1203, 1138 cm^-1^

**AMM** (ESI) *m/z* 505.1772 [calcd for C_23_H_27_ClFN_6_O_2_ (M+H)^+^ 505.1766]

**[α]**_D_^23^ = + 9 ° (*c* 0.2, MeOH)

**Synthesis of MCG-III-051**


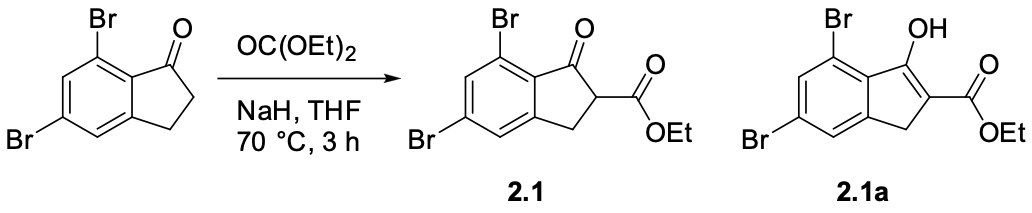


*Tautomers* **2.1** and **2.1a.** To a stirred suspension of sodium hydride (60% dispersion in mineral oil, 82.8 mg, 2.07 mmol) and diethyl carbonate (1.3 mL, 10. mmol) in THF (3 mL) in a sealed microwave vial at room temperature under N_2_ atmosphere was added dropwise a solution of 5,7- dibromo-1*H*-indanone (500. mg, 1.72 mmol) in THF (5.6 mL). The reaction mixture was heated to 70 °C for 3 h, then cooled and diluted with EtOAc and H_2_O and neutralized with aq. 1N HCl. Layers were separated and the resulting aqueous layer was extracted with EtOAc. The combined organic layers were washed with sat. aq. NaCl and dried over anhydrous MgSO_4_ then concentrated *in vacuo* to afford the desired product as an off-white solid (588 mg, 94% yield). The product was isolated as a mixture of keto and enol tautomers (ratio 2:1 by ^1^H-NMR).

**^1^H NMR** (500 MHz, Chloroform-*d*): keto tautomer δ 7.72 (s, 1H), 7.61 (s, 1H), 4.24 (q, *J* = 6.9 Hz,

2H), 3.74 (dd, *J* = 8.6, 4.2 Hz, 1H), 3.50 (dd, *J* = 17.7, 4.3 Hz, 1H), 3.28 (dd, *J* = 17.5, 8.4 Hz,

1H), 1.31 (t, *J* = 7.2 Hz, 3H); enol tautomer δ 10.76 (broad s, 1H), 7.68 (s, 1H), 7.53 (s, 1H), 4.32

(q, *J* = 7.2 Hz, 2H), 3.47 (s, 2H), 1.36 (t, *J* = 7.2 Hz, 3H);

**^13^C NMR** (126 MHz, CDCl_3_) δ 195.41, 169.05, 168.25, 157.17, 147.04, 135.58, 134.39, 134.26,

131.65, 130.30, 128.76, 127.02, 123.61, 120.98, 116.05, 103.77, 99.95, 61.99, 60.48, 53.92,

32.21, 29.01, 14.35, 14.14;

**IR** (ATR) ν_max_ 3300, 2983, 1653, 1418, 1243, 1018, 771 cm^-1^;

**AMM** (ESI) *m/z* 359.9018 [calcd for C_12_H_10_Br_2_O_3_ (M+H)^+^ 359.8997].


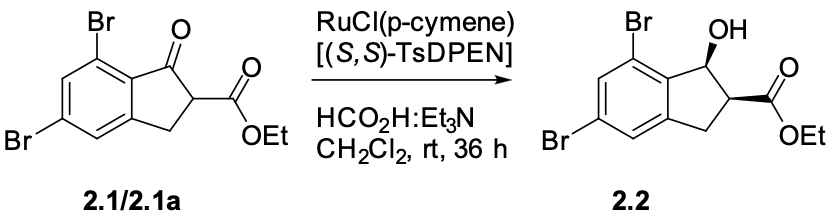


b*-Hydroxyester* **2.2.** To a stirred solution of tautomers **2.1** and **2.1a** (92.9 mg, 0.257 mmol) and RuCl(p-cymene)[(*S*,*S*)-TsDPEN] (3.3 mg, 0.005 mmol) in CH_2_Cl_2_ (2.5 mL) at room temperature under N_2_ atmosphere was added formic acid:triethyl amine complex 5:2 (0.03 mL, 0.5 mmol). The reaction mixture was stirred at room temperature for 36 h, then diluted with sat. aq. NaHCO_3_. Layers were separated and the resulting aqueous layer was extracted with CH_2_Cl_2_. The combined organic layers were washed with sat. aq. NaCl and dried over anhydrous MgSO_4_ then concentrated *in vacuo*. Flash column chromatography (SiO_2_, 80:20 hexanes:EtOAc) afforded the product as a colorless oil (0.085 g, 91% yield).

**[**α**]_D_**^23^ +21.82 (c. 0.21, CH_2_Cl_2_);

**^1^H NMR** (500 MHz, CDCl_3_): ^1^H NMR (500 MHz, Chloroform-*d*) δ 7.55 (s, 1H), 7.36 (s, 1H), 5.38

(dd, *J* = 5.8, 4.6 Hz, 1H), 4.26 (q, *J* = 7.1 Hz, 2H), 3.58 (dd, *J* = 16.3, 9.8 Hz, 1H), 3.35 (ddd, *J* =

9.9, 8.3, 5.9 Hz, 1H), 3.11 (dd, *J* = 16.4, 8.3 Hz, 1H), 2.77 (d, *J* = 4.6 Hz, 1H), 1.33 (t, *J* = 7.1 Hz,

3H);

**^13^C NMR** (126 MHz, CDCl_3_) δ 171.52, 146.13, 141.37, 132.92, 127.23, 123.54, 120.76, 75.35,

61.17, 48.92, 33.34, 14.24;

**IR** (thin film, KBr) ν_max_ 3460, 2929, 1730, 1216, 1038, 853 cm^-1^;

**AMM** (ESI) m/z 384.9057 [calcd for C_12_H_12_Br_2_O_3_Na (M+Na)^+^ 384.9051].


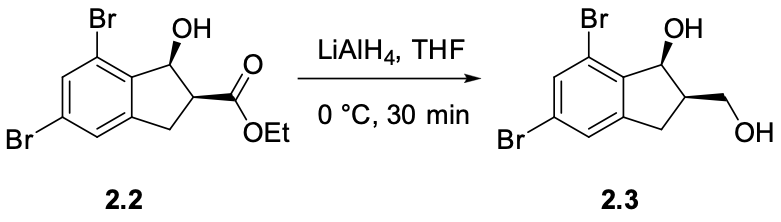


*Diol* **2.3**. To a precooled (0 °C) solution of **2.2** (373 mg, 1.02 mmol) in THF (5.1 mL) under N_2_ atmosphere was added portion wise lithium aluminum hydride (31 mg, 0.82 mmol). The reaction mixture was stirred for 30 min at 0 °C then quenched with sat. aq. sodium potassium tartrate and stirred at room temperature for 10 min. The quenched solution was diluted with H_2_O then EtOAc. The layers were separated, and the resulting aqueous layer was extracted with EtOAc. The combined organic layers were washed with sat. aq. NaCl dried over MgSO_4_ and concentrated *in vacuo*. Flash column chromatography (SiO_2_ 20:80 hexanes:EtOAc) afforded the product as a white solid (285 mg, 86% yield).

**[**α**]_D_**^23^ +25.21 (c. 0.15, CH_2_Cl_2_);

**^1^H NMR** (500 MHz, Chloroform-*d*) δ 7.53 (s, 1H), 7.36 (s, 1H), 5.34 (dd, *J* = 6.7, 2.9 Hz, 1H), 4.07

– 3.99 (m, 1H), 3.97 – 3.84 (m, 1H), 3.17 (dd, *J* = 16.4, 9.1 Hz, 1H), 2.94 (dd, *J* = 16.4, 8.3 Hz,

1H), 2.76 – 2.64 (m, 1H), 2.57 – 2.43 (m, 2H);

**^13^C NMR** (126 MHz, CDCl_3_) δ 147.61, 142.70, 132.45, 127.50, 123.28, 120.37, 77.09, 61.95,

44.40, 33.42.

**IR** (ATR) ν_max_ 3220, 1586, 1557, 1309, 1158, 849, 686, 561 cm^-1^;


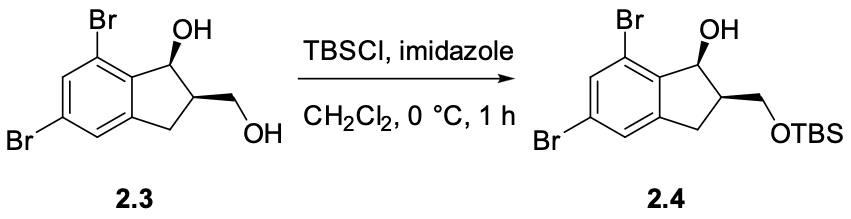


*Silyl ether* **2.4**. To a precooled solution (0 °C) of **2.3** (166 mg, 0.516 mmol) and 1H-imidazole (70.2 mg, 1.03 mmol) in CH_2_Cl_2_ (5 mL) under N_2_ atmosphere was added *tert-*butylchlorodimethylsilane (78 mg, 0.52 mmol). The resulting mixture was stirred at 0 °C for 1 h, then diluted with CH_2_Cl_2_ and aq. sat. NH_4_Cl. The layers were separated and the resulting aqueous layer was extracted with CH_2_Cl_2_. The combined organic layers were washed with sat. aq. NaCl, dried over MgSO_4_, and concentrated *in vacuo*. Flash chromatography (SiO_2_, 80:20 hexanes:EtOAc) afforded the product as a colorless oil (182 mg, 81% yield).

**[**α**]_D_**^23^ +24.15 (c. 0.094, CH_2_Cl_2_);

**^1^H NMR** (500 MHz, Chloroform-*d*) δ 7.53 (s, 1H), 7.33 (s, 1H), 5.24 (t, *J* = 5.1 Hz, 1H), 4.09 –

3.91 (m, 2H), 3.12 (d, *J* = 4.2 Hz, 1H), 3.06 (dd, *J* = 16.2, 9.6 Hz, 1H), 2.90 (dd, *J* = 16.2, 8.0 Hz,

1H), 2.67 – 2.53 (m, 1H), 0.91 (s, 9H), 0.11 (d, *J* = 3.7 Hz, 6H);

**^13^C NMR** (126 MHz, CDCl_3_) δ 147.24, 143.43, 132.56, 127.17, 122.82, 120.85, 76.60, 62.45,

44.78, 33.79, 25.82, 18.17, -5.47, -5.54;

**IR** (ATR) ν_max_ 2952, 2927, 2855, 1590, 1252, 1079, 833, 774 cm^-1^;

**AMM** (ESI) *m/z* 456.9801 [calcd for C_16_H_24_Br_2_O_2_NaSi (M+Na)^+^ 456.9810].


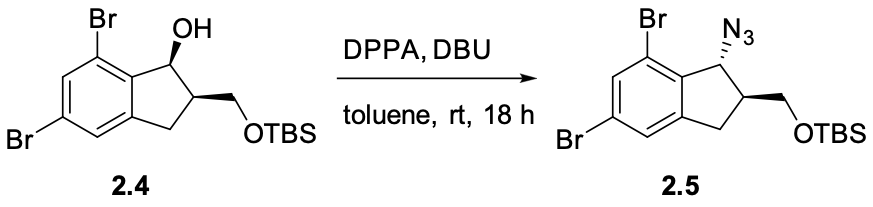


*Azide* **2.5**. To a solution of **2.4** (160. mg, 0.367 mmol) in toluene (1.8 mL) at room temperature under N_2_ atmosphere was added diphenylphosphoryl azide (0.24 mL, 1.1 mmol) then 1,8- diazabicyclo[5.4.0]undec-7-ene (0.16 mL, 1.1 mmol). The resulting mixture was stirred at room temperature for 18 h, then diluted with EtOAc and aq. sat. NH_4_Cl. The layers were separated and the resulting aqueous layer was extracted with EtOAc. The combined organic layers were washed with sat. aq. NaCl, dried over MgSO_4_, and concentrated *in vacuo*. Flash chromatography (SiO_2_, 95:5 hexanes:EtOAc) afforded the product as a colorless oil (124 mg, 78% yield).

**[**α**]_D_**^23^ +19.44 (c. 0.31, CH_2_Cl_2_);

**^1^H NMR** (500 MHz, Chloroform-*d*) δ 7.57 (s, 1H), 7.32 (s, 1H), 4.78 (s, 1H), 3.68 (dd, *J* = 10.3, 5.6

Hz, 1H), 3.47 (t, *J* = 8.8 Hz, 1H), 3.25 (dd, *J* = 17.5, 8.7 Hz, 1H), 2.68 (d, *J* = 13.8 Hz, 2H), 0.86 (s,

10H), 0.04 (d, *J* = 3.7 Hz, 6H);

**^13^C NMR** (126 MHz, CDCl_3_) δ 147.11, 139.01, 133.04, 127.33, 123.37, 121.40, 68.76, 63.86,

48.04, 34.03, 25.75, 18.15, -5.44, -5.50;

**IR** (ATR) ν_max_ 2953, 2929, 2856, 2096, 1559, 1395, 1106, 814, 687 cm^-1^;

**AMM** (ESI) *m/z* 430.9930 [calcd for C_16_H_23_Br_2_ONSi (M-N_2_) 430.9916].


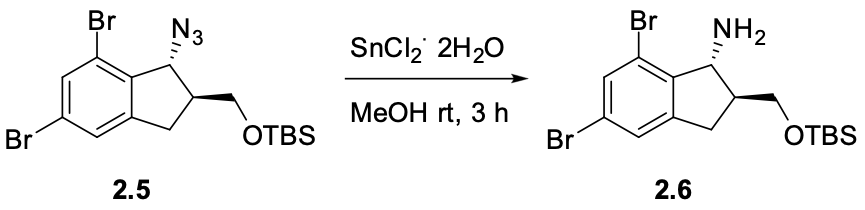


*Amine* **2.6**. To a precooled solution of **2.5** (500. mg, 1.08 mmol) in MeOH (2 mL) under N_2_ atmosphere was added dropwise a solution of tin (II) chloride (366 mg, 1.63 mmol) in MeOH (3.5 mL). The resulting mixture was stirred at room temperature for 3 h, then concentrated *in vacuo*. The resulting solid was taken up in CH_2_Cl_2_ and H_2_O, then treated with 1N aq. NaOH to remove emulsions. The layers were separated, and the resulting aqueous phase was extracted with CH_2_Cl_2_. The combined organic layers were washed with sat. aq. NaCl dried over MgSO_4_ and concentrated *in vacuo* to afford the product as a white solid (433 mg, 92% yield).

**[**α**]_D_**^24^ +56.85 (c. 0.11, CH_3_OH);

**^1^H NMR** (500 MHz, Chloroform-*d*) δ 7.47 (s, 1H), 7.27 (s, 1H), 4.19 (d, *J* = 3.8 Hz, 1H), 3.61 (d, *J*

= 6.5 Hz, 2H), 3.18 (dd, *J* = 16.8, 8.2 Hz, 1H), 2.70 (dd, *J* = 16.8, 4.8 Hz, 1H), 2.41 – 2.28 (m, 1H),

1.87 (s, 2H), 0.85 (s, 9H), 0.03 (s, 6H);

**^13^C NMR** (126 MHz, CDCl_3_) δ 146.52, 144.62, 132.51, 127.34, 121.64, 120.38, 64.56, 60.14,

50.15, 33.70, 25.81, 18.20, -5.41, -5.45;

**IR** (ATR) ν_max_ 2951, 2927, 2855, 1587, 1557, 1252, 1102, 834, 748 cm^-1^;

**AMM** (ESI) *m/z* 434.0139 [calcd for C_11_H_26_N_3_O_3_Br_2_Si (M+H)^+^ 434.0110].


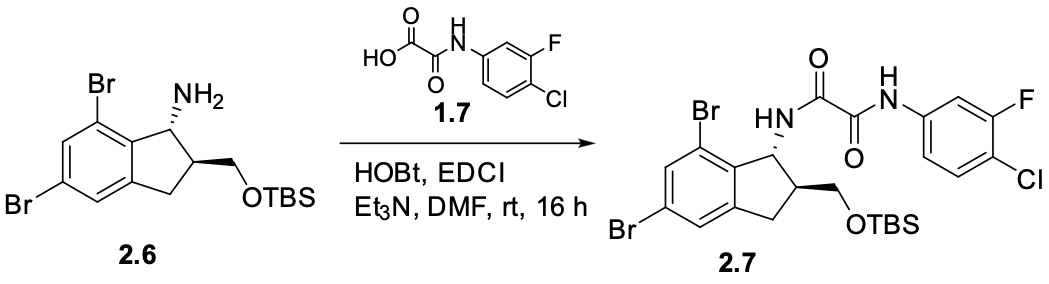


*Oxalamide* **2.7**. To a solution of **2.6** (693 mg, 1.50 mmol), **1.7** (339 mg, 1.50 mmol), 1-hydroxybenzotriazole hydrate (785 mg, 3.61 mmol), and N-(3-dimethylaminopropyl)-N’-ethylcarbodiimide hydrochloride (691 mg, 3.61 mmol) in DMF (7.5 mL) at room temperature under N_2_ atmosphere was added triethylamine (0.96 mL, 6.9 mmol). The resulting mixture was stirred at room temperature for 16 h, then concentrated *in vacuo*. The crude resident was taken up in EtOAc and quenched with sat. aq. NH_4_Cl. The layers were separated and the resulting aqueous phase was extracted with EtOAc. The combined organic layers were washed with H_2_O then sat. aq. NaCl, dried over Na_2_SO_4_, and concentrated *in vacuo*. Flash chromatography (SiO_2_, 90:10 hexanes:EtOAc) afforded the product as a white solid (779 mg, 82% yield).

**[**α**]_D_**^23^ +3.04 (c. 0.10, CH_2_Cl_2_);

**^1^H NMR** (500 MHz, Chloroform-*d*) δ 9.38 (s, 1H), 7.75 – 7.66 (m, 2H), 7.52 (s, 1H), 7.36 (t, *J* =

8.3 Hz, 1H), 7.34 (s, 1H), 7.26 – 7.22 (m, 1H), 5.23 (dd, *J* = 9.0, 4.0 Hz, 1H), 3.75 – 3.64 (m, 2H),

3.27 (dd, *J* = 16.9, 8.4 Hz, 1H), 2.85 (dd, *J* = 16.8, 4.8 Hz, 1H), 2.64 – 2.53 (m, 1H), 0.83 (s, 9H),

0.04 (d, *J* = 3.8 Hz, 6H);

**^13^C NMR** (126 MHz, CDCl_3_) δ 159.12, 158.75, 157.36, 157.14, 147.74, 138.88, 136.30, 136.22,

133.18, 130.82, 127.33, 123.32, 120.75, 117.24, 117.10, 115.95, 115.92, 108.53, 108.32, 64.13,

58.32, 48.74, 34.13, 25.73, 18.15, -5.49;

**IR** (ATR) ν_max_ 3279, 2928, 2855, 1667, 1593, 1517, 1154, 1110, 835, 665 cm^-1^;

**AMM** (ESI) *m/z* 630.9836 [calcd for C_24_H_27_N_2_O_3_FClBr_2_Si (M-H)^-^ 630.9830].


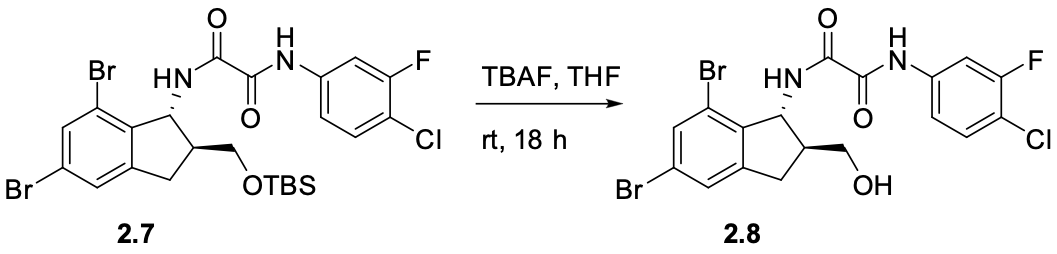


*Alcohol* **2.8**. To a solution of **2.7** (432 mg, 0.680 mmol) in THF (6.8 mL) at room temperature under N_2_ atmosphere was added tetra-*n*-butylammonium fluoride (1 M solution in THF, 2.0 mL, 2.0 mmol). The resulting mixture was stirred at room temperature for 18 h, then diluted with EtOAc and quenched with H_2_O. The layers were separated and the resulting aqueous phase was extracted with EtOAc. The combined organic layers were washed with sat. aq. NaCl, dried over Na_2_SO_4_, and concentrated *in vacuo*. Flash chromatography (SiO_2_, 50:50 hexanes:EtOAc) afforded the product as a white solid (467 mg, 88% yield).

**[**α**]_D_**^23^ +95.19 (c. 0.059, DMSO);

**^1^H NMR** (500 MHz, DMSO-*d*6) δ 11.06 (s, 1H), 9.45 (d, *J* = 9.0 Hz, 1H), 7.95 (d, *J* = 11.8 Hz, 1H),

7.74 (d, *J* = 8.9 Hz, 1H), 7.64 – 7.52 (m, 2H), 7.49 (s, 1H), 5.19 (dd, *J* = 9.2, 5.0 Hz, 1H), 4.80 (t, *J*

= 5.2 Hz, 1H), 3.52 – 3.40 (m, 2H), 3.25 (dd, *J* = 16.8, 8.6 Hz, 1H), 2.76 (dd, *J* = 16.7, 6.0 Hz, 1H);

**^13^C NMR** (126 MHz, DMSO) δ 158.75, 157.70, 155.75, 148.81, 140.19, 138.24, 138.16, 131.79,

130.50, 127.02, 121.50, 120.06, 117.21, 114.34, 114.19, 108.45, 108.25, 62.16, 56.95, 48.32,

33.91;

**IR** (ATR) ν_max_ 3259, 1662, 1591, 1512, 1426, 857, 800, 746 cm^-1^;

**AMM** (ESI) *m/z* 516.8973 [calcd for C_18_H_13_N_2_O_3_FClBr_2_ (M-H)^-^ 516.8965].


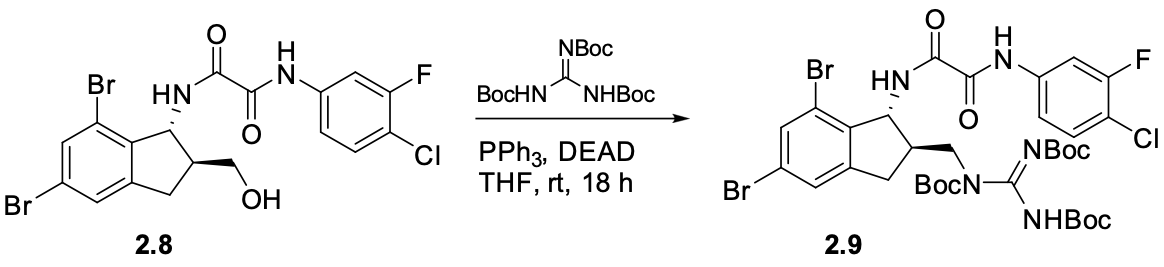


*Common Intermediate* **2.9**. To a precooled (0 °C) solution of **2.8** (287 mg, 0.551 mmol), N,N’,N’’-triBocguanidine (198 mg, 0.551 mmol), and triphenylphosphine (217 mg, 0.827 mmol) in THF (11 mL) under N_2_ atmosphere was added dropwise a diethylazidocarboxylate (0.13 mL, 0.83 mmol). The resulting mixture was stirred at room temperature 18 h, then diluted with EtOAc and quenched with H_2_O. The layers were separated and the aqueous phase was extracted with EtOAc. The combined organic layers were washed with sat. aq. NaCl, dried over Na_2_SO_4_, and concentrated *in vacuo*. Flash chromatography (SiO_2_, 80:20 hexanes:EtOAc) afforded the product as a white solid (338 mg 71% yield).

**[**α**]D**23 +49.65 (c. 0.08, CHCl_3_);

**^1^H NMR** (500 MHz, DMSO-*d*6) δ 11.04 (s, 1H), 10.20 (s, 1H), 9.50 (d, *J* = 8.7 Hz, 1H), 7.94 (dd, *J*

= 11.7, 2.4 Hz, 1H), 7.72 (dd, *J* = 8.9, 2.3 Hz, 1H), 7.61 (s, 1H), 7.58 (t, *J* = 8.7 Hz, 1H), 7.48 (s,

1H), 4.98 (dd, *J* = 8.7, 4.9 Hz, 1H), 3.77 – 3.58 (m, 2H), 2.74 – 2.63 (m, 1H), 1.49 – 1.32 (m, 9H);

**^13^C NMR** (126 MHz, DMSO) δ 158.85, 158.62, 158.26, 157.75, 155.81, 152.52, 150.44, 148.23,

147.38, 146.37, 139.51, 138.30, 138.22, 131.99, 130.60, 127.03, 121.64, 120.12, 117.30, 117.27,

114.42, 114.28, 108.51, 108.30, 82.14, 81.05, 79.07, 58.01, 49.34, 45.77, 34.96, 27.86, 27.76,

27.67, 27.62;

**IR** (ATR) ν_max_ 3275, 2980, 1664, 1514, 1368, 1244, 1121, 762 cm^-1^;

**AMM** (ESI) *m/z* 860.1066 [calcd for C_34_H_42_N_5_O_8_FClBr_2_ (M+H)^+^ 860.1073].


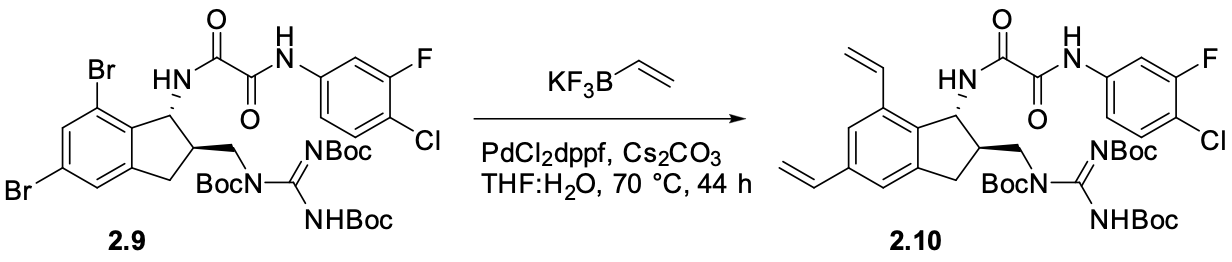


*Divinyl* **2.10**. To a flask charged with **2.9** (200. mg, 0.232 mmol), potassium vinyltrifluoroborate (93 mg, 0.70 mmol), [1,1’-bis(diphenylphosphino)ferrocene]dichloropalladium (II) (19 mg, 0.023 mmol) and cesium carbonate (454 mg, 1.39 mmol) at room temperature under N_2_ atmosphere was added THF (10 mL) and H_2_O (1.5 mL). The flask was affixed with a reflux condenser and backfilled and purged with N_2_ (4x) then the reaction mixture was heated to 70 °C and stirred for 44 h. The reaction mixture was allowed to cool then quenched with H_2_O and diluted with EtOAc. The layers were separated, and the resulting aqueous layer was extracted with EtOAc. The combined organic layers were washed with aq. sat. NaCl, dried over Na_2_SO_4_, and concentrated *in vacuo*. Flash chromatography (SiO_2_, 90:10 hexanes:EtOAc) afforded the product as a white solid (82 mg, 47% yield).

**[**α**]_D_**^23^ +36.87 (c. 0.025, CH_2_Cl_2_);

**^1^H NMR** (500 MHz, Chloroform-*d*) δ 10.41 (broad s, 1H), 9.35 (s, 2H), 7.67 (t, *J* = 8.8 Hz, 2H),

7.40 – 7.32 (m, 2H), 7.24 – 7.17 (m, 2H), 6.78 – 6.60 (m, 2H), 5.73 (dd, *J* = 31.0, 17.6 Hz, 2H),

5.40 (dd, *J* = 8.7, 3.6 Hz, 1H), 5.27 (dd, *J* = 11.0, 4.7 Hz, 2H), 4.00 – 3.85 (m, 2H), 3.26 (dd, *J* =

16.5, 8.0 Hz, 1H), 2.90 – 2.78 (m, 1H), 2.69 (dd, *J* = 16.5, 4.2 Hz, 1H), 1.52 – 1.43 (m, 27H);

**^13^C NMR** (126 MHz, CDCl_3_) δ 158.99, 158.49, 157.24, 157.02, 153.32, 152.00, 143.89, 138.76,

136.71, 136.44, 136.34, 136.26, 135.32, 133.12, 130.70, 122.30, 121.76, 116.96, 116.82, 116.41,

115.81, 114.34, 108.37, 108.17, 83.48, 56.89, 49.81, 46.61, 34.58, 29.65, 28.03, 27.96, 27.89;

**IR** (ATR) ν_max_ 3267, 1661, 1607, 1514, 1368, 1257, 1139, 763 cm^-1^;

**AMM** (ESI) *m/z* 754.3037 [calcd for C_38_H_46_N_5_O_8_FCl (M-H)^-^ 754.3019].


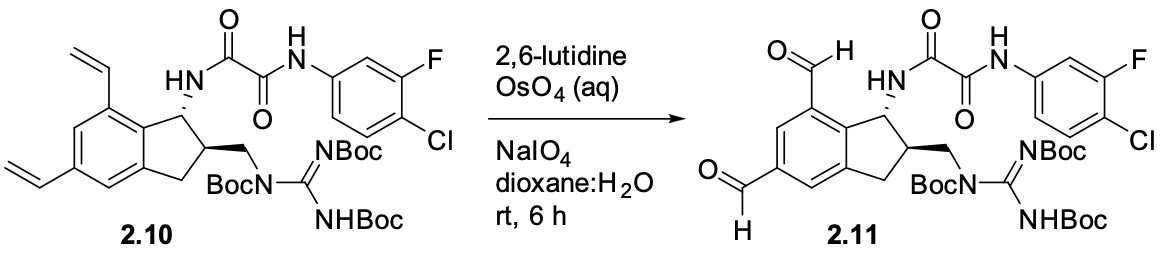


*Dialdehyde* **2.11**. To a precooled (0 °C) of **2.10** (81 mg, 0.11 mmol) in 1,4-dioxane (1.6 mL) and H_2_O (0.5 mL) under N_2_ atmosphere was added osmium tetroxide (0.5% in H_2_O, 0.22 mL, 0.004 mmol) sodium periodate (182 mg, 0.861 mmol) and 2,6-lutidine (0.05 mL, 0.4 mmol). The resulting heterogeneous mixture was allowed to warm to room temperature and vigorously stirred for 6 h, then quenched with H_2_O and diluted with CH_2_Cl_2_. The layers were separated and the resulting aqueous layer was extracted with CH_2_Cl_2_. The combined organic layers were washed with aq. sat. NaCl, dried over Na_2_SO_4_, and concentrated *in vacuo*. Flash chromatography (75:25 hexanes: EtOAc) afforded the desired product as a white solid (43 mg, 52% yield).

**[**α**]_D_**^22^ +70.68 (c. 0.074, CH_2_Cl_2_);

**^1^H NMR** (500 MHz, Chloroform-*d*) δ 10.18 (s, 1H), 10.09 (s, 1H), 9.18 (s, 1H), 8.23 (s, 1H), 8.11

(d, *J* = 8.0 Hz, 1H), 8.03 (s, 1H), 7.65 (dd, *J* = 10.7, 2.5 Hz, 1H), 7.34 (t, *J* = 8.3 Hz, 2H), 7.14 (d,

*J* = 8.7 Hz, 1H), 5.77 (dd, *J* = 8.1, 3.4 Hz, 1H), 4.01 (dd, *J* = 14.2, 6.6 Hz, 1H), 3.80 (dd, *J* = 14.1,

9.2 Hz, 1H), 3.50 (dd, *J* = 16.9, 8.2 Hz, 1H), 3.04 (s, 1H), 2.82 (dd, *J* = 17.2, 3.8 Hz, 2H), 1.48 (d,

*J* = 19.8 Hz, 27H);

**^13^C NMR** (126 MHz, CDCl_3_) δ 190.81, 190.37, 159.12, 158.84, 157.20, 157.15, 153.54, 153.09,

147.15, 146.88, 137.64, 136.65, 136.38, 136.30, 133.56, 131.79, 130.87, 130.43, 120.26, 117.18,

117.04, 115.96, 108.50, 108.29, 83.86, 77.16, 57.48, 49.96, 46.04, 34.68, 28.28, 28.16, 28.07,

28.02, 24.51;

**IR** (ATR) ν_max_ 3460, 2921, 2850, 1730, 1591, 1558, 1450, 1397, 1061, 897, 850 cm^-1^;

**AMM** (ESI) *m/z* 760.2785 [calcd for C_36_H_44_N_5_O_10_FCl (M+H)^+^ 760.2761].


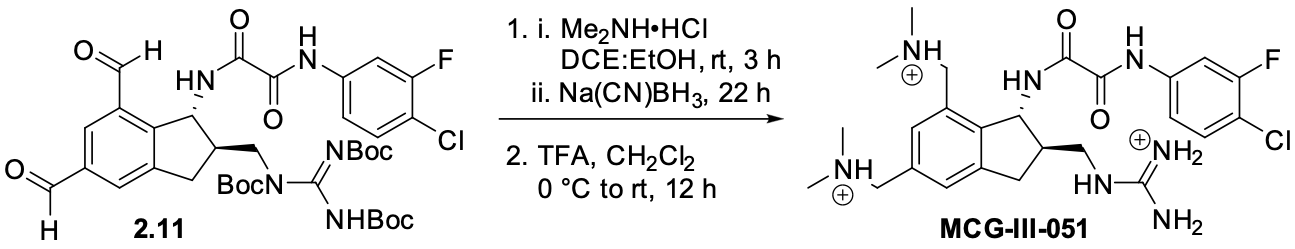


*MCG-III-051*. To a flask charged with **2.11** (42 mg, 0.056 mmol), dimethylamine hydrochloride (91 mg, 1.1 mmol) at room temperature under nitrogen atmosphere was added dichloroethane (2 mL) and ethanol (2 mL). The reaction mixture was stirred at room temperature for 3 h before addition of sodium cyanoborohydride (7.0 mg, 0.11 mmol). The resulting reaction mixture was stirred at room temperature for 22 h, then cooled to 0 °C and quenched with aq. sat. NaHCO_3_ and diluted with CH_2_Cl_2_. The biphasic solution was then adjusted to pH 10 with aq. 1M NaOH and stirred for 30 min. The layers were then separated and the resulting aqueous layer was extracted with CH_2_Cl_2_. The combined organic layers were washed with aq. sat. NaCl, dried over Na_2_SO_4_, and concentrated *in vacuo*. The reside was run through a plug of silica gel, eluting with 20:80 hexanes:EtOAc containing 1% triethylamine, then concentrated *in vacuo* and used in the next step without further purification. The residue was taken up in CH_2_Cl_2_ and cooled to 0 °C before addition of trifluoroacetic acid (0.09 mL, 0.8 mmol). The solution was stirred for 12 h at room temperature, then cooled to 0 °C before addition of trifluoroacetic acid (0.05 mL, 0.6 mmol). The solution was stirred at room temperature for 24 h, then concentrated *in vacuo* and taken up in H_2_O/CH_3_CN (80:20) and subjected to HPLC purification. Conditions: eluent H_2_O/CH_3_CN (80:20 to 50:50, linear gradient); flow rate: 15 mL/min; run time: 15 min. Product-containing fractions were combined and the resulting solution was deep-frozen (-78 °C bath) and lyophilized to afford product as a white powder (6 mg, 12% yield from **1.23**).

**1H NMR** (500 MHz, Acetone-*d*6) δ 10.29 (s, 1H), 9.19 – 9.05 (m, 1H), 7.97 (dd, *J* = 11.6, 2.4 Hz,

1H), 7.81 (s, 1H), 7.74 – 7.69 (m, 1H), 7.61 (s, 1H), 7.51 (t, *J* = 8.6 Hz, 1H), 5.73 (dd, *J* = 8.9, 3.3

Hz, 1H), 4.53 (dd, *J* = 27.9, 13.1 Hz, 2H), 4.40 (d, *J* = 13.2 Hz, 2H), 3.64 – 3.47 (m, 2H), 3.44 –

3.29 (m, 1H), 2.99 (s, 2H), 2.81 (dd, *J* = 16.9, 3.9 Hz, 1H).

**AMM** (ESI) *m/z* 518.2444 [calcd for C_25_H_34_N_7_O_2_FCl (M-2H)^-^ 518.2447]
